# Tolerance and responsive gene expression of *Sogatella furcifera* under extreme temperature stresses are altered by its vectored plant virus

**DOI:** 10.1038/srep31521

**Published:** 2016-08-17

**Authors:** Donglin Xu, Ting Zhong, Wendi Feng, Guohui Zhou

**Affiliations:** 1 Guangdong Province Key Laboratory of Microbial Signals and Disease Control, College of Agriculture, South China Agricultural University, Guangzhou, Guangdong 510642, China

## Abstract

*Southern rice black-streaked dwarf virus* (SRBSDV), a newly emerged fijivirus causing great loss to rice production in eastern and southeastern Asian countries in recent years, is efficiently transmitted by a rice pest, white-backed planthopper (WBPH, *Sogatella furcifera*) in a persistent, circulative propagative manner and can be considered as an insect virus. In this study, SRBSDV infection in WBPH was found to increase the vector’s death rate under extreme cold stress but improve its survival rate under extreme heat stress. Digital gene expression profiling based on RNA-Seq revealed different gene regulation patterns in WBPH under viral and/or temperature stress. Under cold stress, the virus infection upregulated 1540 genes and downregulated 131 genes in the insect, most of which were related to membrane properties and biological processes of actin and cytoskeleton; whereas under heat stress, it upregulated 363 genes and downregulated 548 genes, most of which were associated to metabolism and intracellular organelles. Several types of stress-responsive genes involving intestinal mucin, cuticle protein, ubiquitin protease, immune response, RNA interference and heat shock response, were largely upregulated under cold stress, but largely downregulated under heat stress, by SRBSDV infection. Our results suggest two distinct mechanisms of virus-altered vector insect tolerance to temperature stress.

Interactions between microorganisms and their hosts are ubiquitous in the ecosystem, where their mutualistic or parasitic relationship is established. For insect hosts, they may suffer detrimental effects caused by infection of various pathogens or benefit from their endosymbionts that provide necessary nutrient substances for them and confer them resistance to biotic or abiotic stresses^1^,^2^,^3^. Many insects that feed on plants are vectors of a variety of plant viruses, and studies have shown that plant viruses are not only able to cause pathological changes in their vectors, but also can improve the adaptability of their insect hosts^4^,^5^,^6^,^7^. On the background of complicated plant-virus-vector interactions, the influences of plant viruses on temperature tolerance of their vectors might affect geographic distribution and perniciousness of the pests, spread of viruses, and epidemic of viral plant diseases, and therefore, have extensive ecological importance. However, the effects of plant viruses in this aspect remain poorly studied.

*Sogatella furcifera* (the white-backed planthopper, WBPH) is a devastating rice pest and the only known transmitting vector of *Southern rice black-streaked dwarf virus* (SRBSDV), a newly emerged fijivirus species (family *Reoviridae*) which, since its outbreak in 2009, has overspread to vast areas and imposed a great threat to rice production in eastern and southeastern Asia^8^,^9^. Occurrence and distribution of the disease caused by SRBSDV is highly coincided with the spread and migration of viruliferous WBPH^9^. Annually, the insect populations overwinter in Vietnam and the southern provinces (Hainan and Yunnan) of China, migrate northward over 1,000 km to northern China, Japan and Korea in spring season via the southwest monsoon, and return to their overwintering regions in late August when the monsoon direction shifts^9^,^10^. Although WBPH may overwinter at around 5 °C^11^, the long-distance migratory habit of the insect indicates its susceptibility to both cold and hot climates.

SRBSDV can also be considered as an insect virus because of its ability to propagate and circulate in its vector^9^,^12^. Previous studies have revealed that infection of WBPH by the virus may cause a series of influences on the insect’s biology and behavior, including prolonged nymph period, decreased fecundity, shortened adult lifespan, and altered host selection which, along with its high efficiency of SRBSDV transmission, explained the rapid spread of the virus within a short period of time^9^,^13^,^14^,^15^. In our preliminary study, SRBSDV-induced effects on the insect’s life parameters were temperature-dependent, and the viruliferous vector showed shortened longevity and higher death rates at suboptimal low temperature, and varied survival rates at suboptimal high temperature, as compared to the non-viruliferous insects^13^. In view of the importance of WBPH temperature adaptability to epidemic of SRBSDV, a better understanding of how the virus infection affects the vector’s tolerance to extreme temperature stresses will provide further insight into the ecological effects of this virus and the dynamic of the rice disease. In this study, we report the virus-induced alteration on WBPH acclimation under extreme temperature stresses, and explore the potential mechanisms through comparative transcriptome analysis.

## Results

### Impact of SRBSDV infection on WBPH tolerance to extreme temperature stress

**Figure 1:**
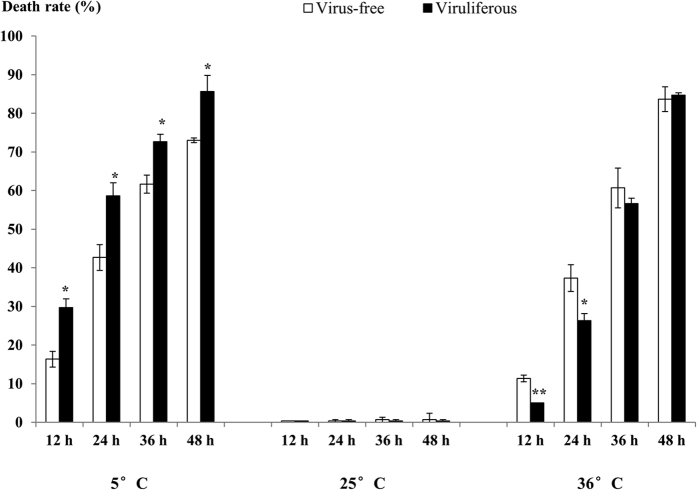
Death rates (%) of 4–5 instar WBPH nymphs at different time points after temperature treatment (the single and double asterisks indicate significance levels of 0.05 and 0.01 respectively).

Compared to the virus-free insects, the SRBSDV-infected WBPH had significantly greater death rates during the 48-h cold treatment (5 °C), but significantly lower death rates under the heat stress (36 °C) for 12 h and 24 h (Fig. 1). However, no difference of death rate was observed between the viruliferous and non-viruliferous insects under suitable temperature (25 °C). It is indicated that SRBSDV infection led to decreased cold tolerance, but improved heat tolerance, of the vector.

### Global changes of WBPH gene expression profile induced by SRBSDV infection and/or temperature stress

**Figure 2:**
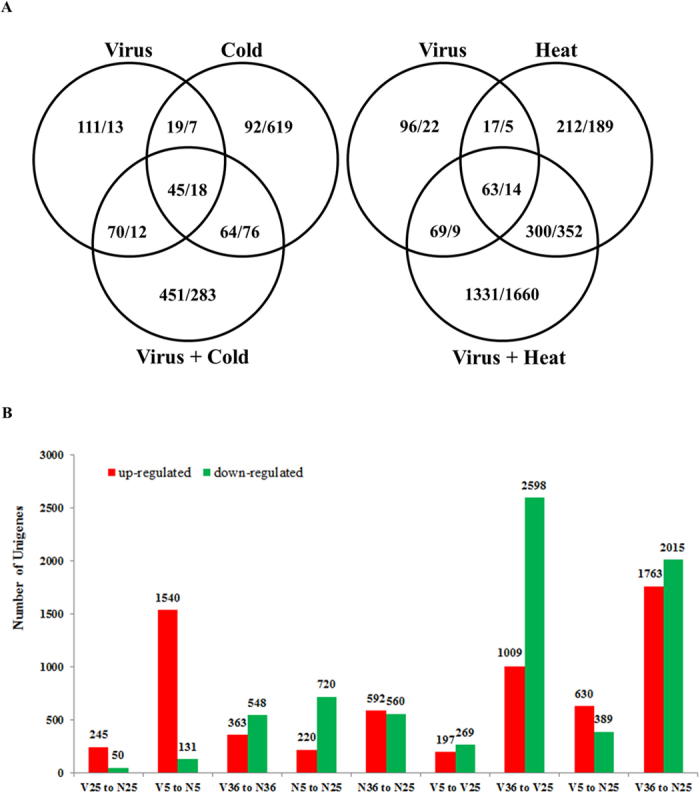
(**A**) Venn diagram of numbers of differentially expressed Unigenes induced by virus infection under cold stress and heat stress (shown as “number of upregulated genes/number of downregulated genes”). (**B**) The quantities of regulated Unigenes in pairwise comparisons. Each sample name indicates its infection status (V = Viruliferous, N = Non-viruliferous) and treating temperature (5, 25 and 36) in Celsius degree.

With the digital gene expression (DGE) sequencing data obtained from the six samples (Table 1), comparative analyses between different samples were carried out according to the criteria of differential expression (FDR < 0.001 and |log_2_Ratio| > 1). The results indicated that 245, 220 and 592 genes were upregulated, while 50, 720 and 560 genes downregulated, under virus infection, cold stress or heat stress, respectively, as compared to the CK (non-viruliferous, 25 °C) (Fig. 2a). SRBSDV infection combined with temperature stress (V5 and V36) induced much greater alterations in gene expression profile than the single stressors (V25, N5, and N36). When under virus infection combined with cold stress (V5), 1019 genes were differentially expressed (630 upregulated and 389 downregulated), while 3778 were altered in expression levels (1763 upregulated and 2015 downregulated) under virus infection combined with heat stress (V36). The majority of these regulated genes, 72.0% (734/1019) from V5 and 79.2% (2991/3778) from V36, were not differentially expressed under any single stressor (Fig. 2A).

Under cold stress and heat stress, the virus infection regulated 1671 genes (1540 upregulated and 131 downregulated; V5 vs. N5) and 911 genes (363 upregulated and 548 downregulated; V36 vs. N36), respectively, considerably greater than that at suitable temperature (245 upregulated and 50 downregulated; V25 vs. N25) (Fig. 2B). This suggested that virus-induced alteration of insect gene expression profile is temperature-dependent and enhanced by temperature stresses. On the other hand, with SRBSDV infection, cold stress induced regulation of 466 genes (V5 vs. V25), less than half of the number (940 genes) observed under the virus-free condition (N5 vs. N25). Heat stress imposed significantly larger influence on gene regulation (3607 genes regulated, V36 vs. V25), as compared to without infection (1152 genes regulated; N36 vs. N25). It is suggested that SRBSDV infection might have different interactions with the two temperature stresses experienced by the insect.

### Regulation of stress response-related genes in WBPH induced by single stressors

As responding to the SRBSDV infection at suitable temperature (25 °C), 88 genes were differentially expressed that were distributed in 10 significantly enriched GO terms, including catechol- and phenol-containing compound biosynthetic processes, external encapsulating structure, integral to membrane, structural constituent of cuticle, tyrosine 3-monooxygenase activity and oxidoreductase activity. Among these differentially expressed transcripts, we identified several types of genes related to stress responses (Tables 2 and S1). Fifteen intestinal mucin protein genes were upregulated (with one of them, CL1432.Contig1, confirmed by RT-qPCR) and five genes were downregulated. Insect intestinal mucin proteins are the key materials that form peritrophic membrane, which is believed to be the first effective mechanical barrier to infection by pathogens^16^,^17^. Seven of these intestinal mucin genes, six upregulated and one downregulated, were also annotated to “cuticle protein”, “structural constituent of cuticle” or “cuticular protein precursor”. In addition, 13 more genes, which encode cuticle protein or endocuticle structural glycoprotein, or take part in chitin-based cuticle development, were found upregulated in viruliferous insects (two of them were confirmed by RT-qPCR). This suggests that enhanced expression of insect cuticle genes might be involved in physical defense against virus infection, or virus accumulation in and transmission by the vector^18^. Seven upregulated genes (with Unigene908 confirmed by RT-qPCR) were identified as E3 ubiquitin protein ligase genes. The ubiquitin-protease pathways have been reported to play important roles in antiviral defense via degrading viral proteins. On the other hand, by modifying these pathways, viruses may enhance their replication and propagation^19^. Unigene10443 was involved in “response to stress”, “immune system process”, “apoptotic signaling pathway” and “positive regulation of apoptotic process, and it was upregulated in the viruliferous samples, suggesting that the virus infection might induce programmed cell death via insect immunity. The expression level of Unigene40466 was over four times higher in infected WBPH, as compared to the control. This gene is known to be involved in salivary gland cell autophagy. SRBSDV may replicate and accumulate in the salivary gland of WBPH^20^, and rice reoviruses might manipulate autophagy pathways in vectors to overcome the salivary gland barriers for their successful transmission^21^. Unigene10836 was homologous to a defensin B gene of an insect *Rhodnius prolixus*, suggesting that upregulation of this gene might participate in a defensin-mediated defense response. Upregulated by SRBSDV infection, Unigene47276 was annotated to “mRNA 3′-UTR binding”, and it functions in RNA interference process, a universal virus-resistance mechanism existing in eukaryotic organisms. Two upregulated chemosensory protein genes were annotated to “response to virus”. Additionally, upregulation of four takeout protein (or precursor) genes, two cytochrome P450 genes and one heat shock protein (Hsp) gene induced by the virus infection was observed (with one P450 gene and the Hsp gene confirmed by RT-qPCR). Insect chemosensory proteins can be involved in regulation of immunity^22^ and their takeout proteins are related to feeding behavior, starvation response, and lifespan^23^,^24^,^25^. The heat shock pathway components are closely associated with insect antiviral defense^26^.

Under the cold stress, 52 genes were regulated and they were distributed in 15 enriched GO terms, including chaeta development, protein complex subunit organization, actin filament-based process, pheromone biosynthetic process, lipid particle, structural constituent of cuticle, acyl-CoA desaturase activity, structural molecule activity, retinal binding, myosin binding, and more. Under heat stress, 17 genes in three enriched GO terms (protein polymerization, structural constituent of cuticle, and structural constituent of chitin-based cuticle) were differentially expressed. All types of stress response-related genes responding to SRBSDV infection were found significantly regulated under cold or heat stress. Compared to virus infection, both cold stress and heat stress induced regulation of greater numbers of intestinal mucin genes, cuticle-related genes, and ubiquitin-related genes (Tables 2 and S1). Notably, under cold stress, there were two Hsp genes being upregulated, whereas heat stress upregulated 17 Hsp genes including small Hsps, *Hsp68, Hsp70, Hsp78, Hsp90, Hsp101*, and *Hsp110*, and downregulated a heat shock transcription factor (Hsf). Besides, one upregulated and two downregulated facilitated trehalose transporter genes, one downregulated trehalose 6-phosphate synthase gene (confirmed by RT-qPCR) and two downregulated apolipoprotein D genes were identified upon cold stress, whereas one upregulated and one downregulated facilitated trehalose transporter genes were found under heat stress. Trehalose is known to improve insect tolerance to cold^27^, and apolipoprotein D may increase stress resistance and longevity of insects^28^.

### Regulation of stress response-related genes in WBPH induced by SRBSDV infection combined with temperature stress

Under virus infection combined with cold stress, 26 significantly enriched GO terms containing 78 differentially expressed genes were detected. These GO terms included chitin metabolic process, glucosamine-containing compound metabolic process, amino sugar metabolic process, aminoglycan metabolic process, negative regulation of immune system process, chitin binding, structural constituent of chitin-based cuticle, and more. Relative to only cold stress, the combination of SRBSDV infection and cold stress induced upregulation of dramatically larger quantities of intestinal mucin, cuticle-related, ubiquitin-related, and immune response-related genes, while downregulated fewer of these four types of stress response-related genes (Table 2). Moreover, several RNA interference-related, takeout protein, cytochrome P450, trehalose-related, and apolipoprotein genes were upregulated.

Under SRBSDV infection combined with heat stress, 78 significantly enriched GO terms containing 1162 differentially expressed genes were identified, and more than two thirds of these GO terms were in the biological process category. These GO terms included structural constituent of cuticle, structural constituent of ribosome, metabolic processes of various cellular nutrients, catabolic processes of nuclear-transcribed mRNA and cellular macromolecules, regulation of ubiquitin-protein ligase activity and protein ubiquitination, signal transduction processes, translational elongation and termination, cotranslational protein targeting to membrane, and notably, several viral processes - viral transcription, genome expression, infectious cycle, and reproduction. The viral processes involved 32 differentially expressed genes, only two of which were upregulated with the rest all downregulated. Compared with only heat stress, the combination of SRBSDV infection and heat stress regulated greater amounts of all eleven types of stress resistance-response genes, especially intestinal mucin, cuticle-related, ubiquitin-related, and immune response-related genes (Table 2). It is noteworthy that under the virus-plus-heat stress, 17 Hsp genes were upregulated and seven ones were downregulated, but downregulation of Hsps could not be observed under only heat stress (Supplementary Table S1). This suggests that viral processes may interact or interfere with the insect’s heat shock pathways.

### SRBSDV-induced changes on WBPH gene expression profile under temperature stresses

**Figure 3:**
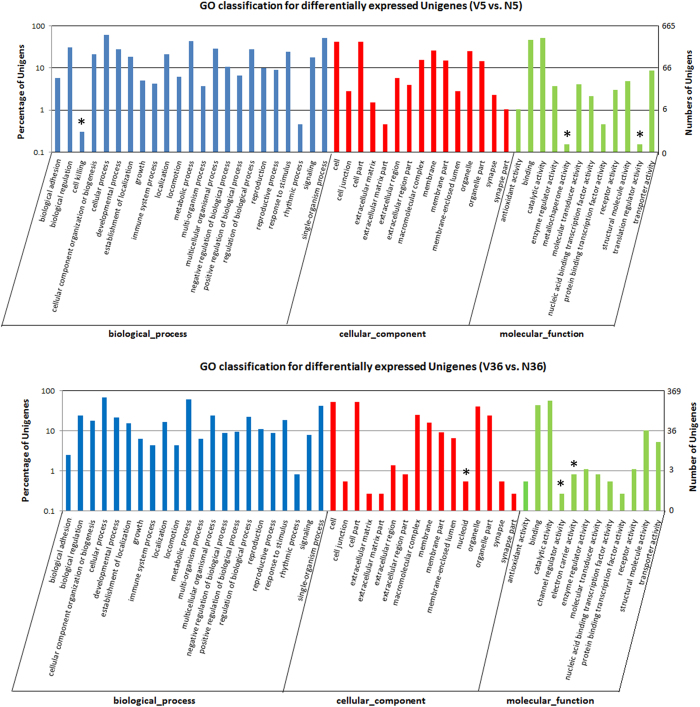
GO classification of SRBSDV-regulated genes under cold (above) and heat (below) stress. Asterisks indicate the subcategories that appear in only one, not both, cases.

To explore the potential mechanisms of the SRBSDV impact on WBPH temperature endurance, we compared the gene expression profiles between the viruliferous and the virus-free insects, under cold and heat stresses, respectively. The results indicated that under cold stress, there were 1540 upregulated and 131 downregulated genes responding to SRBSDV infection; whereas under heat stress, there were 363 upregulated and 548 downregulated genes responsive to the virus infection. GO classification indicated 50 subcategories of genes differentially expressed under each temperature stress, and there was only slight difference in GO subcategory collection between both stresses (Fig. 3). However, the virus-induced gene regulation patterns under different temperature stresses were distinct. GO term Enrichment analysis showed that under cold stress, 283 virus-regulated genes were assigned to 13 enriched GO terms, and the most abundant term was “membrane” (175 genes, 61.8%), followed by “protein complex subunit organization” (50 genes), “actin filament-based process” (41 genes), “actin cytoskeleton organization” (40 genes), “extracellular region” (39 genes), and “cytoskeletal protein binding” (38 genes) (Supplementary Table S2). Under heat stress, 291 virus-regulated genes in 66 enriched GO terms were identified, and the most abundant terms included “metabolic process” (214 genes, 73.5%), “cellular metabolic process” (188 genes), “primary metabolic process” (180 genes), “organic substance metabolic process” (187 genes), “nitrogen compound metabolic process” (108 genes), “cytoplasm” (145 genes), “cytoplasm part” (121 genes), “cell” (190 genes), “cell part” (190 genes), “intracellular” (183 genes), “intracellular part” (177 genes), and “intracellular organelle” (146 genes) (Supplementary Table S2). This finding suggested that under cold stress, SRBSDV infection primarily affects cell membrane properties, as well as actin- and cytoskeleton-related processes and functions, while under heat stress, it largely influences the insect’s metabolism and properties of intracellular organelles.

SRBSDV infection-induced regulation of stress response-related genes in WBPH clearly exhibited different patterns under cold and hot stresses. In total, 156 stress-responsive genes were upregulated by the virus infection but only seven were downregulated, under cold stress. Under heat stress, 83 stress-responsive genes were downregulated but only 25 genes were upregulated by the infection (Table 2). Under cold stress, upregulation of intestinal mucin genes, cuticle-related genes, ubiquitin-related genes and immune response-related genes was dominant, with few of these genes downregulated by the virus infection. However, under heat stress, most of the intestinal mucin genes, ubiquitin-related genes and immune response-related genes responding to SRBSDV infection were downregulated; and seven upregulated and six downregulated cuticle-related genes were identified. Six genes involved in RNA interference, four cytochrome P450 genes and three chemosensory protein genes were more abundantly expressed in virus-infected insects than in virus-free insects under cold stress, while only one gene of each of these three types were downregulated by the virus infection under heat stress. The infection upregulated seven trehalose-related genes and downregulated one under cold stress, while only one facilitated trehalose transporter gene was upregulated and one trehalose 6-phosphate synthase gene was downregulated under heat stress. Elevated expression levels of three apolipoprotein genes in viruliferous insects relative to virus-free ones were detected under cold stress, but no regulation of this kind of genes was found under heat stress. Only one Hsp gene was upregulated by the virus infection under cold stress (Unigene9618, an *Hsp70*, confirmed by RT-qPCR), whereas 10 Hsp genes (eight *Hsp70*, one *Hsp90* and one *Hsp20*) and one Hsf gene were all downregulated under heat stress (with Unigene16177 and Unigene9991, an *Hsp70* and an *Hsp20*, confirmed by RT-qPCR). Furthermore, 15 genes that belong to the enriched GO terms regarding to viral processes (transcription, genome expression, infectious cycle and reproduction) were found downregulated by SRBSDV infection under heat stress, while three genes upregulated by the infection under cold stress were identified as involved in viral transcription, genome replication, and reproduction, respectively.

### RT-qPCR validation for the RNA-Seq data

The twelve stress-responsive Unigenes that encode intestinal mucin, cuticle protein, ubiquitin-protein ligase, stress-activated immune response-related protein, RNA-induced silencing complex component, cytochrome P450, heat shock protein, and trehalose transporter, respectively, exhibited the same regulation orientations in the five comparison sets (virus/CK, cold/CK, heat/CK, infected/uninfected under cold stress, and infected/uninfected under heat stress) in RT-qPCR, as revealed in RNA-Seq, with a few exceptional cases of insignificant regulation (i.e., |log_2_Ratio|<1) (Supplementary Table S3).

## Discussion

In this study, our temperature tolerance experiments established that SRBSDV infection resulted in decreased extreme low temperature tolerance but improved extreme high temperature tolerance of its sole vector, WBPH, a long-distance migratory rice pest. This finding has implications for ecological effects caused by SRBSDV. The overwintering regions of WBPH are limited in warm-temperature tropical and subtropical areas^9^. Weakened resistance of viruliferous vectors to cold temperature may reduce their overwintering areas and effectiveness, and therefore, decrease the primary source of SRBSDV infection in most rice producing areas in China. This inhibition reasonably explains the milder epidemic of the virus after its severe occurrence in 2010^9^. On the other hand, virus-enhanced heat tolerance enables its vector to better survive, propagate and migrate in the warm-weather seasons. The resulted increased viruliferous population, as the secondary source of infection, can aggravate epidemic of the viral rice disease in summer and cause severer plant damage by WBPH. Taken together, the impacts of SRBSDV on temperature stress tolerance of its vector may cause annual fluctuation of occurrence and severity of the disease in China.

Beside the detrimental influences imposed by a plant virus on its vector^13^,^14^, the beneficial effects of plant and/or insect viruses on their hosts have been also well-recorded, including improved fitness or stress resistance of plant hosts, and increased fecundity, longevity, and host suitability of insect vectors^6^,^29^,^30^,^31^,^32^. However, the impacts, harmful or beneficial, of plant virus infection on vector insects’ fitness under biotic or abiotic stress are less recognized. Pusag *et al*. reported that tomato yellow curl virus-infected *Bemisia tabaci* suffered higher mortality than the virus-free whiteflies, both at 4 °C and at 35 °C^33^. Our study provided another example of plant virus-modulated vector stress tolerance, and revealed an interesting difference in modulation manners under cold and heat stresses. This information expands our knowledge of the interplay of a biotic and an abiotic stresses simultaneously. At present, the interactive effects of combined stresses on plants have been extensively described^34^,^35^, but little is known about the scenario in insects, especially in plant virus-transmitting vectors. Our tolerance tests indicated that SRBSDV infection alone did not affect survival of WBPH. In the virus-plus-cold and virus-plus-heat cases, the presence of SRBSDV infection resulted in only an increase of 11~16% and a decrease of 6 ~ 11%, respectively, to death rates of its vector (Fig. 1). It is suggested that temperature exerts the major influence on the vector’s survival under combined stresses (virus infection and temperature). Further investigations on other plant viruses and their vectors may ascertain whether this is a common phenomenon.

Prior to our study, Xu *et al*. published their comparative analysis of gene expression profiles between the SRBSDV-infected and virus-free WBPH. They identified thousands of differentially expressed genes in the viruliferous insects^36^. However, we found only 295 WBPH genes responding to SRBSDV infection, although both studies obtained similar amounts of RNA-Seq data (81,338 vs. 83,876 Unigenes). This huge discrepancy is most likely due to different preparations of insect materials for RNA-Seq. The previous study seemed to use first generation nymphs. In this study, insects were propagated to fourth generation, yielding a population of minimum genetic diversity for comparative analyses, and 36 °C was chosen as the temperature of extreme heat stress, as our preliminary experiment at higher temperatures of 37 °C and 38 °C resulted in total mortality of test insects within 2 hours.

In this study, we present the first report on changes in gene expression profile in insect vector responding to temperature stress and combined virus and temperature stresses. Our results showed that the combined stresses induced or enhanced regulation of diverse immunity processes, and the virus infection combined with heat stress elevated heat shock responses (Tables 2 and S1). Heat shock response is a defensive mechanism existing in cellular organisms that can be triggered by numerous physical or chemical, biotic or abiotic, stimuli. This often involves upregulated expression of the Hsf and a number of Hsps, functioning as molecular chaperones to prevent other proteins from misfolding under stress conditions^26^,^37^,^38^. In addition to its functions in heat tolerance and cold survival of the insect^37^,^38^, the role of heat shock response in antiviral defense has been shown recently in fruit fly, although how this defense works remains unanswered^26^. Insect Hsp genes might be regulated by the RNAi pathway, another mechanism that plays important roles in antiviral defense^39^,^40^ and has been proven to affect survival, infection and transmission of rice reoviruses and their interactions with leafhopper and planthopper vectors^21^,^41^,^42^, and this defense mechanism can also be suppressed by viruses^43^. In the previous study^36^ and this study, SRBSDV has been found to regulate RNAi pathway-related genes (AGO and DCL) in WBPH. It is likely that the intricate crosstalk of heat shock and RNAi pathways might exist in WBPH under virus infection and temperature stresses. In this study, under heat combined with viral stresses, 17 Hsp genes were upregulated but eight ones downregulated, accompanied by downregulation of 30 genes involved in viral transcription, genome expression, reproduction, and infection processes. This indicates antiviral effects of WBPH heat shock response, which in turn, can be inhibited by the virus. A similar suppression by cricket paralysis virus has been observed in fruit fly cells^44^. Several studies have shown that higher temperature can upregulate the expression of RNA silencing components (AGO, RDR and DCL) in plants and thus enhance their defense against plant viruses^45^,^46^,^47^. Although a few RNAi-related genes in WBPH were found upregulated under virus infection combined with heat stress in this study, it is not clear if similar enhancement of antiviral defense occurs in the insect.

The comparison of gene expression profiles between the viruliferous and non-viruliferous WBPH exposed to temperature stresses suggests that the predominant upregulation of gene expression is likely the fundamental cause of reduced cold tolerance of virus-infected insects. Despite the upregulation of almost all stress resistance-related genes (Table 2) and improvement of cell membrane and cytoskeleton properties that may facilitate cold tolerance^38^,^48^, these insects probably died of exhaustion arisen from the global gene upregulation responding to extreme cold stress. In contrast, downregulation of gene expression dominated in the virus-induced alteration under extreme heat stress, including inhibition of many types of stress responsive genes. Notably, all differentially expressed Hsp and Hsf genes were downregulated in the heat-tolerant (viruliferous) insects, as compared to in the virus-free vector, under extreme heat stress. Virus-infected whiteflies have been shown to have higher expression levels of *Hsp40, Hsp70* and *Hsp90* and they survived better under extreme high temperature, as compared to the uninfected ones^33^. The implications of the inhibition of heat shock response in the viruliferous WBPH are unclear. Although the general downregulation (particularly suppression of a series of metabolic processes) and the modification of intracellular organelles might be associated with enhanced heat tolerance of SRBSDV-infected insects, more in-depth studies are required to elaborate the mechanisms behind the virus-regulated vector survivability under temperature stresses.

## Methods

### Culturing of rice plants

Rice cultivar used in this study, TaiChung Native-1 (TN-1, purchased from a local rice seed company) was grown using the water planting method according to Yoshida *et al*.^49^. Rice seeds were soaked in water for 24 h, germinated in an incubator at 30 °C, and then sown in a 2-liter beaker half-filled with culture solution until they grew to three- to four-leaf stage seedlings. A batch of uniformly grown seedlings were selected and transferred to new beakers (each of which contained 20 seedlings and 1.5 L of the culture solution renewed once a week), and then cultured in a growth chamber under the conditions of 25 ± 0.5 °C, RH 75 ± 5%, and 12 h light/12 h dark.

For these seedlings, a part of them (about 50 plants) were inoculated with SRBSDV-carrying third- or fourth-instar WBPH nymphs (five nymphs per seedling on average) propagated from SRBSDV-infected rice plants maintained in our lab. Each beaker with insects and inoculated plants was sealed using a transparent plastic cover with small punctured holes (made via a toothpick) on it for aeration, until manual removal of the insects at 48 h post-inoculation. RT-PCR detection was conducted for each inoculated plant at 15 days after inoculation to confirm virus infection. The confirmed SRBSDV-infected plants and the non-inoculated plants were used for WBPH propagation.

### WBPH propagation

The SRBSDV-free WBPH individuals (confirmed by RT-PCR detection after they died) were originally collected from rice fields in Guangzhou, Guangdong province, China, and defined as first generation. About 30 pairs of male and female insects were matched on healthy rice plants of three-leaf stage. After oviposition and hatching, the obtained nymphs were transferred to new healthy plants and reared there until their eclosion to yield second generation adults. Following the same process, the insects were propagated to fourth generation to generate a population with minimum genetic diversity. A part of the newly hatched, fourth-generation nymphs were transferred to SRBSDV-infected plants for a 48-h feeding, and moved back to healthy plants to allow them to grow into the fourth or fifth instar or adults. The virus-fed insects and the virus-free ones (not been fed on infected plants) were then used for temperature stress tolerance tests. After the tests, the virus-fed and the healthy plant-fed insects were detected by RT-PCR individually to confirm their infection status.

### Tolerance tests for WBPH under temperature stresses

The cold stress and heat stress tests were conducted at 5 °C and 36 °C, respectively, with a parallel test at suitable temperature 25 °C as the control. Each test contained three replicates and each replicate contained 100 viruliferous and 100 virus-free fourth- to fifth-instar nymphs. The insects were kept in nylon gauze-sealed glass tubes (10 individuals per tube) that were incubated under set temperatures. The alive/dead status of the insect individuals at 12 h, 24 h, 36 h and 48 h after the cold or heat treatment was examined by touching them with a brush pen, and the deathlike (i.e., not moving when touched) insects without recovery after restoration at 25 °C for 30 minutes were confirmed as dead. After the experiments were completed, all the tested insects were subjected to virus detection by RT-PCR individually to confirm their infection. Death rates of the viruliferous and the virus-free groups in each treatment were calculated respectively. The percentage data were then subject to arcsine transformation and a t-test was performed by the SPSS19.0 software for statistical significance analysis.

### RNA extraction and RT-PCR detection

Total RNA extraction from rice leaf or WBPH samples was conducted using a TRIzol reagent (TaKaRa, Dalian, China) and RT-PCR detection was done with a one-step RNA PCR Kit (TaKaRa) following the manufacturers’ instructions, as described previously^13^. The thermal cycling conditions were 50 °C for 30 min, 94 °C for 2 min, 35 cycles of 94 °C for 30 sec, 53 °C for 30 sec and 72 °C for 50 sec, followed by a final extension at 72 °C for 5 min.

### Library construction, transcriptome sequencing, and Unigene annotation

Total RNA of WBPH was extracted from a pool sample containing 100 fourth- to fifth-instar nymphs, and treated by DNase I (TaKaRa). Concentration, purity and integrity of the total RNA were verified by an Agilent Bioanalyzer 2000 system (Agilent, CA, USA) before it was used for library construction. The whole process of transcriptome sequencing was performed by Beijing Genome Institute (BGI, Shenzhen, China), including mRNA enrichment by oligo-dT-attached magnetic beads; fragmentation of mRNA; the first strand cDNA synthesis by random hexamer primers; the second strand cDNA synthesis using DNA polymerase I and RNase H; cDNA purification, 3′-end blunting, adenylation and 3′-end adapter addition; size selection of cDNA fragments by the AMPure XP system (Beckham Coulter, Beverly, USA); PCR amplification and product purification; and RNA-Seq by Illumina HiSeq™ 2000. After sequencing, clean reads were obtained by filtering raw data (i.e., removing reads with adaptor sequences, reads with >5% nucleotides as “N”, and those having >20% nucleotides in read with a Q-value ≤10). De novo assembling of clean reads was carried out using Trinity software^50^ to generate gapless contigs, then the reads were mapped back to the contigs, and distances of paired-end reads were determined to provide frames for assembling the contigs into Unigene sequences. The Unigenes were subject to further splicing and gene family clustering via TGICL software, yielding nonredundant Unigenes that were then subject to NT, NR, Swiss-Prot, KEGG and COG annotation with an E-value threshold of 10^−5^. Based on the NR annotation information, Gene Ontology (GO) enrichment analysis using Balst2GO software^51^ and GO functional classification using WEGO software^52^ were performed. The nonredundant Unigenes was used as reference sequences for differential expression profiling analysis.

### Digital gene expression (DGE) profiling

To reveal the differential expression of WBPH genes under temperature and/or virus infection stress, DGE RNA-Seq was performed for six RNA libraries, which contained the total RNA from six WBPH samples, respectively: V25 (viruliferous, reared at 25 °C), N5 (non-viruliferous, cold-treated at 5 °C for 2 h), V5 (viruliferous, cold-treated at 5 °C for 2 h), N36 (non-viruliferous, heat-treated at 36 °C for 2 h), V36 (viruliferous, heat-treated at 36 °C for 2 h), and N25 (non-viruliferous, reared at 25 °C). Every sample consisted of 100 four-to-five-instar nymphs. Total RNA extraction and quality check were carried out as mentioned earlier. The whole DGE sequencing process was carried out by BGI using Illumina HiSeq™ 2000. After sequencing, raw data were processed into clean reads, which then were mapped back to the reference sequences (i.e., the assembled transcriptome sequences) using software SOAPaligner/SOAP2. RPKM (Reads Per kb per Million reads) values of the Unigenes, which represent their expression levels, were calculated by normalization of the numbers of mapped clean reads. Statistical analysis based on fold changes of expression levels was done using software DESeq R package to reveal the differences of gene expression profile between the treated samples and the control. To limit false discovery rate (FDR), P-values were adjusted according to the method described by Benjamini *et al*.^53^. The Unigenes with an FDR value <0.001 and fold change >2 (|log_2_Ratio|>1) were considered as differentially expressed. These differentially expressed Unigenes were subject to NR BLAST, GO functional enrichment analysis (with corrected P-value < 0.05 as the criterion), and KEGG orthology (KO) annotation.

### RT-qPCR validation

Twelve differentially expressed Unigenes identified by RNA-Seq analysis, which represent several types of stress response-related genes, were selected for RT-qPCR validation (Supplementary Table S3). The primers (Supplementary Table S4) were designed based on the transriptome sequencing result. One microgram of the total RNA extracted from the same samples used for RNA-Seq was reverse transcribed using a PrimerScript^TM^ RT Reagent Kit (TaKaRa), followed by a qPCR via a SYBR^®^ Premix Ex Taq^TM^ II kit (TaKaRa) in a Thermal Cycler^®^ Dice Real Time System TP800 (TaKaRa), following the manufacturers’ instructions. The qPCR cycling conditions were 95 °C for 30 sec followed by 40 cycles of 95 °C for 15 sec and 60 °C for 30 sec. After completion of qPCR cycling, the melting curves were generated at 95 °C to verify the specificity of amplification. To evaluate of the quality of qPCR amplification, standard curve analysis was done using a 10-fold serial cDNA dilution for each gene. For sample normalization, the 18S rRNA of WBPH was used as an internal control. All reactions were performed in triplicate and the average Ct values obtained were used in data normalization by the 2^−△△Ct^ method^54^.

## Additional Information

**How to cite this article**: Xu, D. *et al*. Tolerance and responsive gene expression of *Sogatella furcifera* under extreme temperature stresses are altered by its vectored plant virus. *Sci. Rep.* **6**, 31521; doi: 10.1038/srep31521 (2016).

## Supplementary Material

Supplementary Information

## Figures and Tables

**Table 1:** Summary of digital gene expression (DGE) sequencing data derived from six samples

Sample Name	Total bases	Clean reads	Mapped reads	Perfect match	≤2 bp Mismatch	Unique match	Multi-position match
N25 (CK)	616,964,635	12,591,115 (99.51%)	9,705,816 (77.08%)	7,620,464 (60.52%)	2,085,352 (16.56%)	8,193,954 (65.08%)	1,511,862 (12.01%)
V25	581,315,420	11,863,580 (99.48)	9,256,413 (78.02%)	7,175,332 (60.48%)	2,081,081 (17.54%)	7,651,401 (64.49%)	1,605,012 (13.53%)
N5	617,885,443	12,609,907 (99.48%)	9,842,966 (78.06%)	7,632,811 (60.53%)	2,210,155 (17.53%)	8,178,557 (64.86%)	1,664,409 (13.20%)
V5	616,278,096	12,577,104 (99.37%)	9,981,776 (79.36%)	7,768,295 (61.77%)	2,213,481 (17.60%)	8,132,996 (64.67%)	1,848,780 (14.70%)
N36	620,744,299	12,668,251 (99.70%)	9,965,444 (78.66%)	7,644,668 (60.35%)	2,320,776 (18.32%)	8,098,411 (63.93%)	1,867,033 (14.74%)
V36	589,664,285	12,033,965 (99.54%)	9,234,792 (76.74%)	7,059,011 (58.66%)	2,175,781 (18.08%)	7,490,020 (62.24%)	1,744,772 (14.50%)

Each sample name indicates its infection status V = viruliferous, N = non-viruliferous and the Celsius temperature under which it was treated.

**Table 2:** Numbers of upregulated/downregulated stress response-related genes in WBPH responding to SRBSDV infection and/or temperature stress.

Gene species	Stress treated relative to CK					Infected relative to virus-free	
	Virus	Cold	Virus + Cold	Heat	Virus + Heat	5 °C	36 °C
Intestinal mucin	15/5	23/12	57/7	24/17	45/101	25/2	6/32
Cuticle protein or other cuticle-related genes	19/1	27/20	44/5	27/0	46/5	22/1	7/6
Ubiquitin protease or other ubiquitin-related genes	7/0	5/18	18/1	14/10	61/48	59/1	5/21
Immune response-related genes (programmed cell death, autophagy, and apoptotic process, etc.)	3/0	2/4	16/1	7/3	37/32	43/2	9/20
RNA interference-related	1/0	0/1	2/0	1/2	6/7	6/0	0/1
Takeout protein	4/0	4/0	4/0	3/0	7/1	0/0	0/0
Cytochrome P450	2/0	1/1	3/0	4/0	12/0	4/0	0/1
Chemosensory protein	2/0	1/0	3/0	4/0	4/1	3/0	0/1
Heat shock pathway components	1/0	2/0	0/0	17/1	17/9	1/0	0/12
Facilitated trehalose transporter/trehalose 6-phosphate synthase	0/0	1/3	2/0	1/1	4/2	7/1	1/1
Apolipoprotein	0/0	0/2	3/0	0/0	3/0	3/0	0/0
Total	48/5	53/49	129/12	90/33	224/198	156/7	25/83

## References

[b1] Oliver K. M., Degnan P. H., Burke G. R. & Moran N. A. Facultative symbionts in aphids and the horizontal transfer of ecologically important traits. Annual Review of Entomology 55, 247–266 (2010). 10.1146/annurev-ento-112408-085305 19728837

[b2] Feldhaar H. Bacterial symbionts as mediators of ecologically important traits of insect hosts. Ecological Entomology 36, 533–543 (2011).

[b3] Shan H. W., Lu Y. H., Bing X. L., Liu S. S. & Liu Y. Q. Differential responses of the whitefly *Bemisia tabaci* symbionts to unfavorable low and high temperatures. Microbial Ecology 68, 472–482 (2014). 24788211 10.1007/s00248-014-0424-3

[b4] Rubinstein G. & Czosnek H. Long-term association of tomato yellow leaf curl virus with its whitefly vector *Bemisia tabaci*: Effect on the insect transmission capacity, longevity and fecundity. Journal of General Virology 78, 2683–2689 (1997). 9349491 10.1099/0022-1317-78-10-2683

[b5] Inoue T. & Sakurai T. Infection of *Tomato spotted wilt virus* (TSWV) shortens the life span of thelytokous *Thrips tabaci* (Thysanoptera: Thripidae). Applied Entomology and Zoology 41, 239–246 (2006).

[b6] Mckenzie C. L. Effect of *Tomato mottle virus* (ToMoV) on *Bemisia tabaci* biotype B (Homoptera : Aleyrodidae) oviposition and adult survivorship on healthy tomato. Florida Entomologist 85, 367–368 (2002).

[b7] Belliure B., Janssen A. & Sabelis M. W. Herbivore benefits from vectoring plant virus through reduction of period of vulnerability to predation. Oecologia 156, 797–806 (2008). 18392858 10.1007/s00442-008-1027-9 PMC2469278

[b8] Zhou G. H. . Southern rice black-streaked dwarf virus: a new proposed *Fijivirus* species in the family Reoviridae. Chinese Science Bulletin 53, 3677–3685 (2008).

[b9] Zhou G., Xu D., Xu D. & Zhang M. Southern rice black-streaked dwarf virus: a white-backed planthopper-transmitted fijivirus threatening rice production in Asia. Frontiers in Microbiology 4, 270, doi: 10.3389/fmicb.2013.00270 (2013). 24058362 PMC3766826

[b10] Catindig J. L. A. . In Planthoppers: new threats to the sustainability of intensive rice production systems in Asia (ed. Heong K. L. .) 191–220 (IRRI, 2009).

[b11] Hu S.-J. . Projecting distribution of the overwintering population of *Sogatella furcifera* (Hemiptera: Delphacidae), in Yunnan, China with analysis on key influencing climatic factors. Journal of Insect Science 15, 148, doi: 10.1093/jisesa/iev131 (first published online: Oct 22, 2015). 26494777 PMC4622178

[b12] Pu L. . Transmission characters of Southern rice black-streaked dwarf virus by rice planthoppers. Crop Protection 41, 71–76 (2012).

[b13] Tu Z., Ling B., Xu D., Zhang M. & Zhou G. Effects of southern rice black-streaked dwarf virus on the development and fecundity of its vector. Sogatella furcifera. Virology Journal 10, 145, doi: 10.1186/1743-422X-10-145 (2013). 23663428 PMC3698214

[b14] Lei W., Liu D., Li P. & Hou M. Interactive Effects of Southern Rice Black-Streaked Dwarf Virus Infection of Host Plant and Vector on Performance of the Vector, *Sogatella furcifera* (Homoptera: Delphacidae). Journal of Economic Entomology 107, 1721–1727 (2014). 26309259 10.1603/EC13569

[b15] Wang H., Xu D., Pu L. & Zhou G. Southern rice black-streaked dwarf virus alters insect vectors’ host orientation preferences to enhance spread and increase rice ragged stunt virus co-infection. Phytopathology 104, 196–201 (2014). 24047253 10.1094/PHYTO-08-13-0227-R

[b16] Hegedus D., Erlandson M., Gillot, t. C. & Toprak U. New Insights into Peritrophic Matrix Synthesis, Architecture, and Function. Annual Review of Entomology 54, 285–302 (2009). 10.1146/annurev.ento.54.110807.090559 19067633

[b17] Rose M. C. & Voynow J. A. Respiratory tract mucin genes and mucin glycoproteins in health and disease. Physiological Reviews 86, 245–278 (2006). 16371599 10.1152/physrev.00010.2005

[b18] Liu W. . Proteomic analysis of interaction between a plant virus and its vector insect reveals new functions of Hemipteran cuticular protein. Molecular and Cellular Proteomics 14, 2229–2242 (2015). 26091699 10.1074/mcp.M114.046763 PMC4528249

[b19] Glickman M. H. & Ciechanover A. The ubiquitin-proteasome proteolytic pathway: Destruction for the sake of construction. Physiological Reviews 82, 373–428 (2002). 11917093 10.1152/physrev.00027.2001

[b20] Jia D. . Development of an insect vector cell culture and RNA interference system to investigate the functional role of fijivirus replication protein. Journal of Virolology 86, 5800–5807 (2012). 10.1128/JVI.07121-11 PMC3347266 22398296

[b21] Wei T. & Li Y. Rice reoviruses in insect vectors. Annual Review of Phytopathology 2016 May 25. [Epub ahead of print] doi: 10.1146/annurev-phyto-080615-095900 (2016). 27296147

[b22] Picimbon J. F. . Purification and molecular cloning of chemosensory proteins from *Bombyx mori*. Archives of Insect Biochemistry and Physiology 44, 120–129 (2000). 10897093 10.1002/1520-6327(200007)44:3<120::AID-ARCH3>3.0.CO;2-H

[b23] Sarov-Blat L., So W. V., Liu L. & Rosbash M. The *Drosophila takeout* gene is a novel molecular link between circadian rhythms and feeding behavior. Cell 101, 647–656 (2000). 10892651 10.1016/s0092-8674(00)80876-4

[b24] Bauer J. . Comparative transcriptional profiling identifies takeout as a gene that regulates life span. Aging (Albany NY) 2, 298–310 (2010). 20519778 10.18632/aging.100146 PMC2898020

[b25] Chamseddin K. H. . *Takeout*-dependent longevity is associated with altered Juvenile Hormone signaling. Mechanism of Ageing and Development 133, 637–646 (2012). 10.1016/j.mad.2012.08.004 PMC3518612 22940452

[b26] Merkling S. H. . The heat shock response restricts virus infection in *Drosophila*. Scientific Reports 5, 12758, doi: 10.1038/srep12758 (2015). 26234525 PMC4522674

[b27] Benoit J. B., Lopez-Martinez G., Elnitsky M. A., Lee R. E. & Denlinger D. L. Dehydration-induced cross tolerance of *Belgica antarctica* larvae to cold and heat is facilitated by trehalose accumulation. *Comparative Biochemistry and Physiology*. Part A, Molecular & Integrative Physiology 152, 518–523 (2009). 10.1016/j.cbpa.2008.12.009 19141330

[b28] Walker D. W., Muffat J., Rundel C. & Benzer S. Overexpression of a *Drosophila* homolog of apolipoprotein D leads to increased stress resistance and extended lifespan. Current Biology 16, 674–679 (2006). 16581512 10.1016/j.cub.2006.01.057

[b29] Roossinck M. J. The good viruses: viral mutualistic symbioses. Nature Review Microbiology 9, 99–108 (2011). 21200397 10.1038/nrmicro2491

[b30] Stumpf C. F. & Kennedy G. G. Effects of tomato spotted wilt virus isolates, host plants, and temperature on survival, size, and development time of *Frankliniella occidentalis*. Entomologia Experimentalis et Applicata 123, 139–147 (2007).

[b31] Ogada P. A., Maiss E. & Poehling H.-M. Influence of tomato spotted wilt virus on performance and behaviour of western flower thrips (*Frankliniella occidentalis*). Journal of Applied Entomology 137, 488–498 (2013).

[b32] Zheng X., Zhang J., Chen Y., Dong J. & Zhang Z. Effects of *Tomato zonate spot virus* infection on the development and reproduction of its vector *Frankliniella occidentalis* (Thysanoptera: Thripidae). Florida Entomologist 97, 549–554 (2014).

[b33] Pusag J. C., Hemayet Jahan S. M., Lee K. S., Lee S. & Lee K. Y. Upregulation of temperature susceptibility in *Bemisia tabaci* upon acquisition of *Tomato yellow leaf curl virus* (TYLCV). Journal of Insect Physiology 58, 1343–1348 (2012). 22841829 10.1016/j.jinsphys.2012.07.008

[b34] Ramegowda V. & Senthil-Kumar M. The interactive effects of simultaneous biotic and abiotic stresses on plants: Mechanistic understanding from drought and pathogen combination. Journal of Plant Physiology 176, 47–54 (2015). 25546584 10.1016/j.jplph.2014.11.008

[b35] Suzuki N., Rivero R. M., Shulaev V., Blumwald E. & Mittler R. Abiotic and biotic stress combinations. New Phytologist 203, 32–43 (2014). 24720847 10.1111/nph.12797

[b36] Xu Y., Zhou W., Zhou Y., Wu J. & Zhou X. Transcriptome and comparative gene expression analysis of *Sogatella furcifera* (Horvath) in response to Southern rice black-streaked dwarf virus. PloS One 7, e36238, doi: 10.1371/journal.pone.0036238 (2012). 22558400 PMC3338671

[b37] Feder M. E. & Hofmann G. E. Heat-shock proteins, molecular chaperones, and the stress response: evolutionary and ecological Physiology. Annual Review of Physiology 61, 243–282 (1999). 10.1146/annurev.physiol.61.1.243 10099689

[b38] Rinehart J. P. . Upregulation of heat shock proteins is essential for cold survival during insect diapause. PNAS 104, 11130–11137 (2007). 17522254 10.1073/pnas.0703538104 PMC2040864

[b39] Chiba S. & Suzuki N. Highly activated RNA silencing via strong induction of dicer by one virus can interfere with the replication of an unrelated virus. PNAS 112, E4911–E4918 (2015). 26283371 10.1073/pnas.1509151112 PMC4568273

[b40] Spellberg M. J. & Marr M. T. II FOXO regulates RNA interference in *Drosophila* and protects from RNA virus infection. PNAS 112, 14587–14592 (2015). 26553999 10.1073/pnas.1517124112 PMC4664361

[b41] Lan H. . Small interfering RNA pathway modulates initial viral infection in midgut epithelium of insect after ingestion of virus. Journal of Virology 90, 917–929 (2015). 26537672 10.1128/JVI.01835-15 PMC4702677

[b42] Lan H. . Small interfering RNA pathway modulates persistent infection of a plant virus in its insect vector. Scientific Reports 6, 20699, doi: 10.1038/srep20699 (2016). 26864546 PMC4750021

[b43] Cernilogar F. M. . Chromatin-associated RNA interference components contribute to transcriptional regulation in *Drosophila*. Nature 480, 391–395 (2011). 22056986 10.1038/nature10492 PMC4082306

[b44] Cevallos R. C. & Sarnow P. Temperature Protects Insect Cells from Infection by Cricket Paralysis Virus. Journal of Virology 84, 1652–1655 (2010). 19906924 10.1128/JVI.01730-09 PMC2812312

[b45] Qu F. . RDR6 Has a Broad-Spectrum but Temperature-Dependent Antiviral Defense Role in *Nicotiana benthamiana*. Journal of Virology 79, 15209–15217 (2005). 16306592 10.1128/JVI.79.24.15209-15217.2005 PMC1316014

[b46] Zhang X., Zhang X., Singh J., Li D. & Qu F. Temperature-Dependent Survival of *Turnip Crinkle Virus*-Infected *Arabidopsis* Plants Relies on an RNA Silencing-Based Defense That Requires DCL2, AGO2, and HEN1. Journal of Virology 86, 6847–6854 (2012). 22496240 10.1128/JVI.00497-12 PMC3393596

[b47] Ghoshal B. & Sanfaçon H. Temperature-dependent symptom recovery in *Nicotiana benthamiana* plants infected with tomato ringspot virus is associated with reduced translation of viral RNA2 and requires ARGONAUTE 1. Virology 456-457, 188–197 (2014). 24889238 10.1016/j.virol.2014.03.026

[b48] Lee R. E., Damodaran K., Yi S.-X. & Lorigan G. A. Rapid cold-hardening increases membrane fluidity and cold tolerance of insect cells. Cryobiology 52, 459–463 (2006). 16626678 10.1016/j.cryobiol.2006.03.003

[b49] Yoshida S., Forno D. A., Cock. J. H. & Gomez K. A. Laboratory Manual for Physiological Studies of Rice (3rd ed. International Rice Research Institute, Manila, Philippines) 61–64 (1976).

[b50] Grabherr M. G. . Full-length transcriptome assembly from RNA-Seq data without a reference genome. Nature Biotechnology 29, 644–652 (2011). 10.1038/nbt.1883 PMC3571712 21572440

[b51] Conesa A. . Blast2GO: a universal tool for annotation, visualization and analysis in functional genomics research. Bioinformatics 21, 3674–3676 (2005). 16081474 10.1093/bioinformatics/bti610

[b52] Ye J. . WEGO: a web tool for plotting GO annotations. Nucleic Acids Research (Web Server issue) 34, W293–W297 (2006). 10.1093/nar/gkl031 PMC1538768 16845012

[b53] Benjamini Y. & Yekutieli D. The control of the false discovery rate in multiple testing under dependency. The Annals of Statistics 29, 1165–1188 (2001).

[b54] Livak K. J. & Schmittgen T. D. Analysis of Relative Gene Expression Data Using Real-Time Quantitative PCR and the 2^−ΔΔCT^ Method. Methods 25, 402–408 (2001). 11846609 10.1006/meth.2001.1262

