# Supplementary material for: Tolerance and responsive gene expression of Sogatella furcifera under extreme temperature stresses are altered by its vectored plant virus
Source: Sci Rep. 2016 Aug 17;6:31521. doi: 10.1038/srep31521 (PMC4987581; doi:10.1038/srep31521)
Supplement: Supplementary Information [file srep31521-s1.pdf]

Tolerance and responsive gene expression of *Sogatella furcifera* under extreme temperature stresses are altered by its vectored plant virus

Donglin Xu*, Ting Zhong*, Wendi Feng & Guohui Zhou**

Supplementary Table S1: Regulated Unigenes in response to SRBSDV infection and/or temperature stress

Unigene ID	NR and/or GO Annotation	Stress/CK					Infected/Uninfected	
		V	C	V+C	H	V+H	5℃	36℃
**1. Intestinal mucin**								
CL1432.Contig1	intestinal mucin-2; cuticular protein 6 precursor	3.421	4.662	4.351	4.388	4.289		
Unigene50913	intestinal mucin-2	2.001		2.501		1.237	2.464	2.327
Unigene50348	intestinal mucin-2	1.905	1.411	1.301	1.659	1.908		
CL2844.Contig2	intestinal mucin-2	1.787	1.325	1.749	1.025	1.32		
CL3235.Contig1	intestinal mucin-2	1.739		2.219	-1.083		2.600	2.051
Unigene50000	intestinal mucin-2; structural constituent of cuticle	1.618		1.555		1.178		
CL3355.Contig2	intestinal mucin-2; structural constituent of cuticle	1.536		1.55		1.265		
CL5261.Contig1	intestinal mucin-2; cuticular protein PxutCPR67Bd	1.318	1.282	1.267				
Unigene45906	intestinal mucin-2	1.206			1.273	1.071		
Unigene22536	intestinal mucin-2	1.189						
CL5261.Contig2	intestinal mucin-2; cuticle protein 18.6, isoform B	1.12	1.144	1.182		1.068		
Unigene24768	intestinal mucin-2	1.093		1.034	1.263			
Unigene44439	intestinal mucin-2	1.092	1.085	1.326		1.172		
CL4229.Contig2	intestinal mucin-2; cuticular protein RR-2 motif 67 precursor	1.041	1.316	1.044		1.038		
Unigene45907	intestinal mucin-2	1.004				1.159		
CL1474.Contig4	intestinal mucin-2	-4.946	-3.042	-9.086	-5.028	-3.33		
CL1404.Contig2	intestinal mucin-2; cuticular protein RR-1 motif 32	-1.886	-1.146	-1.021		-1.265		
CL3841.Contig1	intestinal mucin-2	-1.548	-1.119			-2.159		-1.310
CL1669.Contig1	intestinal mucin-2	-1.057						
CL1669.Contig3	intestinal mucin-2	-1.001		-1.32		-1.693		-1.261
Unigene46706	intestinal mucin-2		1.852	2.011	1.483	1.918		
CL3403.Contig2	intestinal mucin-2		1.74					
CL264.Contig1	intestinal mucin-2		1.73	2.051	1.41	1.898		
CL1474.Contig3	intestinal mucin-2; cuticular protein 23 precursor		1.542	1.29	1.017			
CL1474.Contig5	intestinal mucin-2; cuticular protein 18 precursor		1.474	1.271	1.068	1.192		
CL1474.Contig6	intestinal mucin-2		1.442	1.242	1.044			
Unigene48284	intestinal mucin-2		1.348	1.243	1.184	1.169		
CL264.Contig2	intestinal mucin-2		1.269	1.068	1.36	1.301		
Unigene50015	intestinal mucin-2		1.262	1.392		1.104		
CL1474.Contig1	intestinal mucin-2		1.257					
Unigene629	intestinal mucin-2; cuticular protein CPR71		1.167	1.146				
CL4229.Contig1	intestinal mucin-2; cuticular protein PpolCPR83		1.159	1.243	1.013	1.215		

Unigene50603	intestinal mucin-2; similar to cuticular protein 1, RR-2 family (AGAP001664-PA)		1.159	1.369		1.437		
Unigene50604	intestinal mucin-2; similar to cuticular protein 1, RR-2 family (AGAP001664-PA)		1.136		1.121			
CL1525.Contig1	intestinal mucin-2		1.116	1.328				
CL2126.Contig1	intestinal mucin-2; cuticular protein 43 precursor		1.025			1.167		
Unigene40083	intestinal mucin-2		-1.742				1.963	
Unigene50612	intestinal mucin-2		-1.542					
Unigene51892	intestinal mucin-2		-1.451				1.495	
CL312.Contig3	intestinal mucin-2		-1.255			-1.025		
CL2061.Contig2	intestinal mucin-2		-1.213				1.167	
Unigene46027	intestinal mucin-2		-1.209				1.375	
CL797.Contig3	intestinal mucin-2; cuticular protein		-1.052			-1.824		-1.238
Unigene44330	intestinal mucin-2		-1.036				1.221	
Unigene47399	intestinal mucin-2		-1.021				1.685	1.793
Unigene45759	intestinal mucin-2; cuticular protein 58 precursor			1.118	1.154	1.586		
Unigene1489	intestinal mucin-2; structural constituent of chitin-based cuticle			1.224	1.05	1.583	1.620	
Unigene47066	intestinal mucin-2; structural constituent of chitin-based cuticle				1.001			
Unigene214	intestinal mucin-2				1.476	1.161		
CL797.Contig2	intestinal mucin-2; cuticular protein				1.37			
Unigene51102	intestinal mucin-2			1.496	1.286	1.439		
Unigene48850	intestinal mucin-2			1.497	1.136	1.442		
Unigene923	intestinal mucin-2			1.557	1.099	1.364		
Unigene50514	intestinal mucin-2			1.191	1.074			
Unigene312	intestinal mucin-2			1.489	1.005	1.207		
Unigene40013	intestinal mucin-2				-4.627			
Unigene54476	intestinal mucin-2				-2.305	-2.433		
Unigene15302	intestinal mucin-2				-2.263			
Unigene17985	intestinal mucin-2				-1.858	-2.523		
Unigene35590	intestinal mucin-2				-1.791	-2.218		
CL132.Contig1	intestinal mucin-2				-1.586	-2.447		
Unigene8404	intestinal mucin-2				-1.457	-2.576		-1.119
Unigene22075	intestinal mucin-2				-1.339	-2.368		
Unigene16150	intestinal mucin-2				-1.267	-2.218		
Unigene52670	intestinal mucin-2				-1.248	-1.877		
Unigene10519	intestinal mucin-2				-1.244	-2.131		
Unigene53567	intestinal mucin-2				-1.055	-1.78		
Unigene52879	intestinal mucin-2				-1.028	-1.554		
Unigene16063	intestinal mucin-2				-1.011	-3.429		
Unigene53107	intestinal mucin-2				-1.009	-1.749		-2.418
Unigene42983	intestinal mucin-2			2.282			1.343	
Unigene46934	intestinal mucin-2			2.14			2.330	
Unigene31305	intestinal mucin-2			1.913		1.537	1.975	
Unigene46526	intestinal mucin-2			1.865		1.358		
Unigene34579	intestinal mucin-2			1.822		1.433	1.126	
Unigene1274	intestinal mucin-2			1.809		1.082	1.219	
Unigene46725	intestinal mucin-2			1.773				
Unigene50710	intestinal mucin-2			1.773			1.168	

CL4524.Contig1	intestinal mucin-2			1.645			1.421	
Unigene52055	intestinal mucin-2			1.606		1.431		
Unigene51458	intestinal mucin-2			1.345				
Unigene46862	intestinal mucin-2			1.342			1.947	
Unigene49423	intestinal mucin-2			1.315		1.303		
Unigene22817	intestinal mucin-2			1.302		1.469		
Unigene52069	intestinal mucin-2			1.302		1.008		
CL1498.Contig1	intestinal mucin-2; structural constituent of cuticle			1.261				
Unigene822	intestinal mucin-2			1.233		1.129		
Unigene1269	intestinal mucin-2			1.211		1.263		
Unigene49999	intestinal mucin-2			1.18		1.042		
Unigene33601	intestinal mucin-2			1.168				
Unigene49028	intestinal mucin-2			1.15			1.265	
Unigene49741	intestinal mucin-2			1.113				
Unigene39433	intestinal mucin-2			1.095				
Unigene21913	intestinal mucin-2			1.06			1.276	
Unigene814	intestinal mucin-2; cuticular protein precursor			1.03				
Unigene10003	intestinal mucin-2			1.02		1.292		
CL294.Contig4	intestinal mucin-2			-1.2		-2.217	-1.516	-1.638
CL2267.Contig2	intestinal mucin-2			-1.106		-1.545		-1.441
Unigene52241	intestinal mucin-2			-1.081			-1.147	
CL4302.Contig1	intestinal mucin-2			-1.055				
CL2809.Contig2	intestinal mucin-2					2.589		
Unigene34721	intestinal mucin-2					1.492		
Unigene34757	intestinal mucin-2					1.212	1.396	1.001
Unigene33152	intestinal mucin-2					1.201		1.302
Unigene50514	intestinal mucin-2					1.195		
Unigene29363	intestinal mucin-2					1.042		
Unigene15198	intestinal mucin-2					-4.263		
Unigene57454	intestinal mucin-2					-4.118		
Unigene30043	intestinal mucin-2					-2.986		
Unigene25305	intestinal mucin-2					-2.97		
Unigene17702	intestinal mucin-2					-2.958		
Unigene20214	intestinal mucin-2					-2.871		
Unigene28054	intestinal mucin-2					-2.797		
Unigene18320	intestinal mucin-2					-2.652		
Unigene15108	intestinal mucin-2					-2.593		-2.735
Unigene30038	intestinal mucin-2					-2.407		
Unigene53936	intestinal mucin-2					-2.394		
Unigene17586	intestinal mucin-2					-2.373		
Unigene9179	intestinal mucin-2					-2.311		-1.449
Unigene15035	intestinal mucin-2					-2.297		
Unigene8752	intestinal mucin-2					-2.277		
Unigene16965	intestinal mucin-2					-2.273		
Unigene52654	intestinal mucin-2					-2.214		-1.663
Unigene9153	intestinal mucin-2					-2.192		
Unigene19545	intestinal mucin-2					-2.163		
Unigene18122	intestinal mucin-2					-2.156		
Unigene336	intestinal mucin-2					-2.151		

Unigene53461	intestinal mucin-2					-2.102		
CL3045.Contig1	intestinal mucin-2					-2.093		
Unigene22798	intestinal mucin-2					-2.085		
Unigene41985	intestinal mucin-2					-2.084		
Unigene13741	intestinal mucin-2					-2.08		
CL3725.Contig2	intestinal mucin-2					-2.053		-1.433
Unigene12194	intestinal mucin-2					-2.04		
Unigene23802	intestinal mucin-2					-2.04		
Unigene23829	intestinal mucin-2					-2.03		
Unigene9010	intestinal mucin-2					-2.028		
Unigene28679	intestinal mucin-2					-2.023		
Unigene38188	intestinal mucin-2					-2.019		-1.407
Unigene17728	intestinal mucin-2					-1.945		
Unigene8272	intestinal mucin-2					-1.941		-1.135
CL3085.Contig2	intestinal mucin-2					-1.925		
Unigene24243	intestinal mucin-2					-1.898		
Unigene10654	intestinal mucin-2					-1.889		-1.163
Unigene29213	intestinal mucin-2					-1.857		
Unigene22696	intestinal mucin-2					-1.846		
Unigene30892	intestinal mucin-2					-1.823		
Unigene54713	intestinal mucin-2					-1.803		
Unigene12906	intestinal mucin-2					-1.802		
Unigene23830	intestinal mucin-2					-1.736		
Unigene21890	intestinal mucin-2					-1.714		
Unigene10041	intestinal mucin-2					-1.705		-1.270
Unigene14766	intestinal mucin-2					-1.703		
Unigene19156	intestinal mucin-2					-1.703		
CL112.Contig1	intestinal mucin-2					-1.684		-1.294
Unigene53790	intestinal mucin-2					-1.678		
Unigene52881	intestinal mucin-2					-1.678		
Unigene23495	intestinal mucin-2					-1.669		
Unigene33552	intestinal mucin-2					-1.664		-1.014
Unigene22351	intestinal mucin-2					-1.652		
Unigene54722	intestinal mucin-2					-1.616		
Unigene52805	intestinal mucin-2					-1.592		
CL1025.Contig1	intestinal mucin-2					-1.591		
Unigene21577	intestinal mucin-2					-1.59		
Unigene23711	intestinal mucin-2					-1.565		-1.144
Unigene38665	intestinal mucin-2					-1.543		
Unigene53092	intestinal mucin-2					-1.52		-1.219
Unigene44744	intestinal mucin-2					-1.51		
CL1404.Contig3	intestinal mucin-2; cuticular protein RR-1 motif 32					-1.503		-1.349
Unigene53320	intestinal mucin-2					-1.493		-1.373
Unigene8415	intestinal mucin-2					-1.415		
Unigene48996	intestinal mucin-2					-1.401		-1.053
Unigene9434	intestinal mucin-2					-1.344		
Unigene12727	intestinal mucin-2					-1.344		
Unigene52850	intestinal mucin-2					-1.344		-1.230
Unigene10261	intestinal mucin-2					-1.325		-1.292
CL4302.Contig2	intestinal mucin-2					-1.322		-1.077

CL3374.Contig1	intestinal mucin-2					-1.268		
Unigene53120	intestinal mucin-2					-1.231		
Unigene53297	intestinal mucin-2					-1.212		
CL1060.Contig1	intestinal mucin-2					-1.165		
Unigene52684	intestinal mucin-2					-1.151		
Unigene30791	intestinal mucin-2					-1.102		
CL1576.Contig1	intestinal mucin-2					-1.101		
Unigene9775	intestinal mucin-2					-1.095		-1.088
Unigene9687	intestinal mucin-2; ubiquitin thiolesterase					-1.006		-1.137
Unigene49366	intestinal mucin-2						1.745	
Unigene49124	intestinal mucin-2						1.634	
Unigene49612	intestinal mucin-2						1.148	
CL2410.Contig1	intestinal mucin-2						1.137	
Unigene50854	intestinal mucin-2						1.001	
Unigene1032	intestinal mucin-2							1.071
Unigene28660	intestinal mucin-2							-1.915
Unigene26451	intestinal mucin-2							-1.724
Unigene53141	intestinal mucin-2							-1.396
Unigene33507	intestinal mucin-2							-1.127
Unigene8407	intestinal mucin-2							-1.010

2. Cuticle protein or cuticle related biological process

CL1786.Contig1	cuticle protein	8.804	9.013	8.329	8.39	8.07		
CL1786.Contig2	cuticle protein	7.661	8.209	8.097	7.827	7.499		
Unigene49129	similar to cuticular protein 62Bc	2.064	3.086	2.028	1.733	2.877	-1.058	1.144
Unigene42988	endocuticle structural glycoprotein SgAbd-1-like	2.046	2.553		2.276			
Unigene49670	regulation of adult chitin-containing cuticle	1.85		2.16	1.143	1.956	1.791	
CL2516.Contig2	cuticular protein PxutCPR34	1.684	2.575	2.504	1.55	2.568		1.018
Unigene47693	structural constituent of cuticle	1.154				1.782		
Unigene49998	similar to Larval cuticle protein A3A (TM-A3A)	1.093	1.048	1.324				
Unigene51556	chitin-based cuticle development	1.092				1.366		
CL4080.Contig1	Endocuticle structural glycoprotein SgAbd-8	1.088				1.603		
Unigene1345	cuticular protein 43 precursor	1.07	1.65	1.558	1.166	1.707		
Unigene914	cuticular protein PpolCPH19	1.026				1.178		1.285
Unigene52440	cuticular protein analogous to peritrophins 1-D precursor	1.009						
Unigene92	cuticular protein CPG12-like precursor		1.648	1.529	1.516	1.683		
Unigene13312	cuticular protein 66Cb		1.565	1.332	1.124			
Unigene48631	structural constituent of cuticle		1.401	1.566	1.01	1.448		
CL2948.Contig1	endocuticle structural glycoprotein SgAbd-1		1.292	1.035		1.264		
Unigene39111	pupal cuticle protein		1.24	1.163		1.223		
Unigene48976	structural constituent of chitin-based cuticle		1.169		1.074			
Unigene49888	structural constituent of cuticle		-1.743				1.059	
CL2948.Contig2	endocuticle structural glycoprotein SgAbd-1		-1.131	-1.362		-2.004		-2.512
Unigene233	Cuticle protein 18.6, isoform B		-1.251	-1.514		-1.126		
Unigene34664	cuticular protein 16 precursor		1.013	1.34		1.252		
Unigene48190	cuticular protein PpolCPR68		-1.665	-1.118		-1.348		

CL110.Contig1	cuticle pattern formation; ecdysone-mediated induction of salivary gland cell autophagic cell death (GO:0035072)		-1.641			1.605	2.275	1.531
Unigene40045	cuticle hydrocarbon biosynthetic process (GO:0006723); regulation of autophagy (GO:0010506)		1.025	1.177	1.285	1.952		
Unigene38449	chitin-based larval cuticle pattern formation (GO:0035293)		-2.36				3.059	
Unigene48746	chitin-based larval cuticle pattern formation (GO:0035293)		-1.078					
Unigene33891	larval chitin-based cuticle development (GO:0008363); chitin-based cuticle sclerotization (GO:0007593); ecdysis, chitin-based cuticle (GO:0018990)		-1.006					
CL284.Contig1	cuticle hydrocarbon biosynthetic process (GO:0006723)		-2.412					
CL284.Contig2	cuticle hydrocarbon biosynthetic process (GO:0006723)		-1.43					
CL3282.Contig1	cuticle pattern formation (GO:0035017); E3 ubiquitin-protein ligase UBR3		-1.137				1.178	
Unigene48313	similar to cuticular protein 78				1.000	1.191		
Unigene49664	larval chitin-based cuticle development (GO:0008363); salivary gland cell autophagic cell death (GO:0035071)				1.044	1.144		
Unigene32187	cuticle chitin biosynthetic process (GO:0006035)				1.034	1.273		
Unigene33929	cuticular protein PpolCPG12			1.089	1.077			
CL4694.Contig1	cuticular protein CPG12-like precursor			1.08	1.062			
CL4694.Contig3	cuticular protein CPG12-like precursor			1.14	1.042			
CL1281.Contig2	cuticle protein			2.776		2.615		
Unigene49431	structural constituent of cuticle			1.196				
CL1548.Contig1	similar to cuticular protein 78			1.182		1.2	1.292	
CL221.Contig2	chitin-based embryonic cuticle biosynthetic process (GO:0008362); positive regulation of nurse cell apoptotic process (GO:0045850); antimicrobial humoral response(GO:0019730)			2.119				
CL1402.Contig3	chitin-based embryonic cuticle biosynthetic process (GO:0008362); positive regulation of nurse cell apoptotic process (GO:0045850)			2.115			1.975	
CL221.Contig4	chitin-based embryonic cuticle biosynthetic process (GO:0008362); positive regulation of nurse cell apoptotic process (GO:0045850); antimicrobial humoral response(GO:0019730)			1.651				
CL5285.Contig1	chitin-based embryonic cuticle biosynthetic process (GO:0008362); positive regulation of nurse cell apoptotic process (GO:0045850); antimicrobial humoral response(GO:0019730)			1.536			1.163	
Unigene52987	regulation of adult chitin-containing cuticle pigmentation (GO:0048082); regulation of JAK-STAT cascade (GO:0046425)			1.316			1.115	
Unigene51013	cuticle hydrocarbon biosynthetic process (GO:0006723); regulation of autophagy (GO:0010506)			-1.07				

Unigene43201	cuticular protein RR-2 family member 4 precursor			1.116				
Unigene45880	cuticular protein PpolCPR68			1.044		1.096		
Unigene45269	adult cuticle protein			1.184		1.203		
Unigene51331	pupal cuticle protein 78E			1.137			1.877	
Unigene50068	structural constituent of cuticle			1.089		1.029		
Unigene47692	cuticular protein RR-1 motif 32					1.953		1.230
Unigene9674	structural constituent of cuticle					1.442		
Unigene34623	structural constituent of chitin-based cuticle					1.066		
Unigene24760	molting cycle, chitin-based cuticle (GO:0007591)					3.299		
Unigene51053	chitin-based cuticle development (GO:0040003)					1.492		
Unigene10223	molting cycle, chitin-based cuticle (GO:0007591)					1.383		
Unigene50414	chitin-based cuticle development (GO:0040003)					1.155	1.213	
Unigene46682	chitin-based embryonic cuticle biosynthetic process (GO:0008362)					1.098	1.199	1.145
CL5144.Contig2	molting cycle, collagen and cuticulin-based cuticle (GO:0018996)					-2.678		-2.695
Unigene55668	cuticle development (GO:0042335)					-2.385		
Unigene22689	collagen and cuticulin-based cuticle development (GO:0040002)					-1.903		
Unigene8237	chitin-based larval cuticle pattern formation (GO:0035293)					-1.85		-1.907
Unigene20534	collagen and cuticulin-based cuticle development (GO:0040002)					-1.809		
Unigene10146	molting cycle, collagen and cuticulin-based cuticle (GO:0018996)					-1.168		-1.229
CL1954.Contig1	cuticular protein 23 precursor					1.451		
Unigene48293	Endocuticle structural glycoprotein SgAbd-9					1.254		
CL3238.Contig1	Endocuticle structural glycoprotein SgAbd-2					1.034		
Unigene38448	chitin-based larval cuticle pattern formation (GO:0035293)						4.652	
Unigene59248	structural constituent of cuticle (GO:0042302)						4.256	
CL1994.Contig2	chitin-based larval cuticle pattern formation (GO:0035293)						3.511	
Unigene41899	structural constituent of chitin-based cuticle (GO:0005214)						3.034	1.791
CL5285.Contig11	chitin-based embryonic cuticle biosynthetic process (GO:0008362)						2.763	
CL4555.Contig2	molting cycle, chitin-based cuticle (GO:0007591)						1.906	
Unigene43105	cuticular protein RR-2 motif 143						1.551	
Unigene23515	larval chitin-based cuticle development (GO:0008363)						1.539	
Unigene51541	chitin-based embryonic cuticle biosynthetic process (GO:0008362)						1.359	
**3. Ubiquitin proteasome**								
Unigene52362	E3 ubiquitin-protein ligase MYCBP2	2.725	2.192	2.662	1.662	1.799		
Unigene46500	E3 ubiquitin-protein ligase MYCBP2	1.598		1.118				
Unigene46900	E3 ubiquitin-protein ligase MYCBP2	1.439		1.312				

Unigene908	E3 ubiquitin-protein ligase LRSAM1	1.351		1.625	1.130	1.406	1.743	
Unigene47535	E3 ubiquitin-protein ligase MYCBP2	1.261	1.028	1.495	1.105	1.121		
Unigene40084	E3 ubiquitin-protein ligase MYCBP2	1.199	1.181	1.4				
Unigene51457	E3 ubiquitin-protein ligase RNF31	1.198		1.543		1.229	1.325	
Unigene42370	E3 ubiquitin-protein ligase MYCBP2		1.088			1.158		
CL296.Contig3	ubiquitin carboxyl-terminal hydrolase 25/28		1.018			-1.14		-1.976
Unigene44074	ubiquitin carboxyl-terminal hydrolase 10		-2.752					
Unigene52203	ubiquitin conjugating enzyme 7 interacting protein		-2.630					
CL5030.Contig1	E3 ubiquitin-protein ligase RBBP6		-2.153			1.144	2.593	
Unigene23706	protein ubiquitination (GO:0016567)		-1.954					
Unigene52201	E3 ubiquitin-protein ligase EDD1		-1.763			1.251	1.953	
Unigene51954	E3 ubiquitin-protein ligase BRE1		-1.745				2.467	
Unigene51664	E3 ubiquitin-protein ligase TRIP12		-1.646					
Unigene50510	ubiquitin carboxyl-terminal hydrolase 9/24		-1.522			1.201	2.043	
Unigene43606	ubiquitin carboxyl-terminal hydrolase 25/28		-1.428		-1.767	-1.655		
Unigene1116	ubiquitin thioesterase CYLD; general transcription factor 3C polypeptide 3		-1.408				1.311	
CL1418.Contig1	ubiquitin carboxyl-terminal hydrolase 25/28		-1.336				1.008	
Unigene533	ubiquitin thioesterase CYLD; translation initiation factor 2A		-1.166	-1.043				
Unigene49398	ubiquitin-specific protease activity (GO:0004843); protein deubiquitination (GO:0016579)		-1.153					
Unigene50316	E3 ubiquitin-protein ligase HERC2		-1.065				1.032	
Unigene52200	E3 ubiquitin-protein ligase EDD1		-1.055				1.077	
Unigene33706	ubiquitin ligase complex (GO:0000151); ubiquitin-ubiquitin ligase activity (GO:0034450)		-1.046				1.149	
Unigene47510	E3 ubiquitin-protein ligase MYCBP2			1.784	1.784	2.094		
Unigene23169	ubiquitin carboxyl-terminal hydrolase 25/28				1.683			
Unigene49967	E3 ubiquitin protein ligase 1				1.213	1.235		
CL52.Contig3	ubiquitin C			1.204	1.203	1.891	1.137	
Unigene34044	E3 ubiquitin-protein ligase SH3RF				1.19	1.274		
Unigene20335	E3 ubiquitin-protein ligase SIAH1				1.141	1.027		
Unigene41858	ubiquitin carboxyl-terminal hydrolase 43				1.098			
Unigene46560	ubiquitin-conjugating enzyme E2 H-like; ubiquitin-protein ligase activity (GO:0004842)				1.071	1.307		
Unigene19634	E3 ubiquitin-protein ligase TRIP12				-2.22	-2.915		
CL3950.Contig1	ubiquitin carboxyl-terminal hydrolase; DNA-directed RNA polymerase I subunit RPA2				-1.888	-3.242		
Unigene54112	ubiquitin carboxyl-terminal hydrolase 5/13				-1.839	-2.497		
Unigene23767	E3 ubiquitin-protein ligase HERC3				-1.671	-2.407		
Unigene19360	E3 ubiquitin-protein ligase HUWE1				-1.553	-2.146		
Unigene48951	ubiquitin-activating enzyme E1				-1.208	-1.888		
CL4816.Contig1	ubiquitin carboxyl-terminal hydrolase				-1.133	-1.678		

Unigene41711	SCF ubiquitin ligase complex (GO:0019005); apoptotic process (GO:0006915)			1.395	1.095	1.441	1.241	
Unigene45052	Ubiquitin carboxyl-terminal hydrolase 3				1.095	1.352		
Unigene45480	ubiquitin-like protein 3-like				1.059	1.238	1.319	
Unigene19852	ubiquitin thiolesterase				-2.848	-2.958		
Unigene17438	positive regulation of ubiquitin-protein ligase activity involved in mitotic cell cycle (GO:0051437); negative regulation of ubiquitin-protein ligase activity involved in mitotic cell cycle (GO:0051436); regulation of apoptotic process (GO:0042981); viral reproduction (GO:0016032)				-1.632	-2.172		
Unigene8690	E3 ubiquitin-protein ligase RBBP6			2.198		2.451	1.906	
Unigene8605	ubiquitin carboxyl-terminal hydrolase 36/42			1.451			2.186	
Unigene9879	ubiquitin C; negative regulation of type I interferon production (GO:0032480); egress of virus within host cell (GO:0046788)			1.289		1.721	1.476	
Unigene50028	E3 ubiquitin-protein ligase MYCBP2			1.199				
Unigene49433	E3 ubiquitin-protein ligase MYCBP2			1.185				
Unigene45486	E3 ubiquitin-protein ligase Hakai			1.122		1.167		
Unigene49768	E3 ubiquitin-protein ligase MYCBP2			1.069		1.342		
Unigene49268	anaphase-promoting complex-dependent proteasomal ubiquitin-dependent protein catabolic process (GO:0031145)			1.181			1.324	
Unigene8058	regulation of ubiquitin-protein ligase activity involved in mitotic cell cycle (O:0051439); regulation of gene silencing (GO:0060968);					-1.455		
Unigene46796	ubiquitin-protein ligase activity (GO:0004842); positive regulation of gene silencing by miRNA (GO:2000637); miRNA metabolic process (GO:0010586); protein autoubiquitination (GO:0051865)					1.382		
Unigene34737	E3 ubiquitin-protein ligase RNF103					4.299		
Unigene52198	E3 ubiquitin-protein ligase EDD1					3.884		
Unigene59931	ubiquitin carboxyl-terminal hydrolase 34					2.508		
Unigene33704	ubiquitin conjugation factor E4 B					2.403		
Unigene44886	ubiquitin carboxyl-terminal hydrolase BAP1					2.18		
Unigene13214	E3 ubiquitin-protein ligase HERC4					2.055		
Unigene44415	E3 ubiquitin-protein ligase HERC1					1.803		
Unigene46565	ubiquitin carboxyl-terminal hydrolase 9/24					1.779	1.901	
Unigene20813	deubiquitinating protein VCIP135					1.777		
Unigene1803	E3 ubiquitin-protein ligase TRIP12					1.732		
Unigene41303	E3 ubiquitin-protein ligase EDD1					1.714		
Unigene33707	ubiquitin conjugation factor E4 B					1.649		
Unigene10681	E3 ubiquitin-protein ligase MYCBP2					1.612		
Unigene43300	ubiquitin carboxyl-terminal hydrolase 47					1.546	1.724	
Unigene50358	E3 ubiquitin-protein ligase Topors					1.545		
Unigene51566	E3 ubiquitin-protein ligase UBR2					1.503	1.298	
Unigene21542	E3 ubiquitin-protein ligase MARCH6					1.481	1.665	
Unigene40970	E3 ubiquitin-protein ligase RBBP6					1.473		

Unigene10556	E3 ubiquitin-protein ligase HUWE1					1.451		1.254
Unigene33565	ubiquitin carboxyl-terminal hydrolase 36/42					1.451	1.736	
Unigene47954	ubiquitin-conjugating enzyme E2 O					1.412	1.431	
Unigene46973	ubiquitin carboxyl-terminal hydrolase 6/32					1.391	1.102	
Unigene41265	ubiquitin carboxyl-terminal hydrolase 30					1.329		
Unigene48944	E3 ubiquitin-protein ligase HUWE1					1.271	1.123	
Unigene48436	ubiquitin-conjugating enzyme E2 Q					1.236		
CL2606.Contig1	ubiquitin carboxyl-terminal hydrolase 25/28					1.196		
Unigene48344	homocysteine-responsive endoplasmic reticulum-resident ubiquitin-like domain member 1 protein					1.149	1.039	
Unigene46139	Kip1 ubiquitination-promoting complex protein 2					1.148		
Unigene19889	E3 ubiquitin-protein ligase RNF19A					1.144	1.166	
Unigene8300	E3 ubiquitin-protein ligase SIAH1					1.141		1.524
Unigene47793	E3 ubiquitin-protein ligase NEDD4					1.068		
Unigene48638	E3 ubiquitin-protein ligase RNF13					1.054		
Unigene42249	E3 ubiquitin-protein ligase MUL1					1.027		
CL1107.Contig1	deubiquitinating protein VCIP135					1.02	1.008	
Unigene54946	E3 ubiquitin-protein ligase Praja2					-2.929		
Unigene54974	E3 ubiquitin-protein ligase TRIP12					-2.394		
Unigene22498	ubiquitin carboxyl-terminal hydrolase 10					-2.349		
Unigene28620	ubiquitin carboxyl-terminal hydrolase 7					-2.33		
Unigene8410	ubiquitin-like 1-activating enzyme E1 A					-2.172		
Unigene23221	ubiquitin fusion degradation protein 1					-2.151		
Unigene52944	E3 ubiquitin-protein ligase Topors					-2.129		
Unigene32138	ubiquitin carboxyl-terminal hydrolase 25/28					-2.126		-2.299
CL795.Contig1	ubiquitin carboxyl-terminal hydrolase 25/28					-2.04		
Unigene31413	E3 ubiquitin-protein ligase UBR1					-1.975		
Unigene30563	ubiquitin C					-1.882		-1.425
Unigene14356	ubiquitin-conjugating enzyme E2 variant					-1.728		
Unigene48949	ubiquitin-activating enzyme E1					-1.537		
Unigene50749	ubiquitin carboxyl-terminal hydrolase 25/28					-1.535		-1.437
CL693.Contig3	E3 ubiquitin-protein ligase BRE1					-1.098		
Unigene54151	ubiquitin ligase complex; virus-host interaction (GO:0019048)					-2.08		
Unigene18876	SCF ubiquitin ligase complex (GO:0019005)					-1.097		
Unigene47511	ubiquitin-protein ligase activity (GO:0004842)					1.062		
Unigene48151	E3 ubiquitin-protein ligase KCMF1					1.015		
Unigene13675	polyubiquitin binding (GO:0031593) ; regulation of proteasomal ubiquitin-dependent protein catabolic process (GO:0032434) ; positive regulation of viral genome replication (GO:0045070)					-2.678		
Unigene42330	ubiquitin-protein ligase activity (GO:0004842)					-1.261		
Unigene34975	ubiquitin protein ligase					-2.237		-1.727
Unigene52280	Ubiquitin-conjugating enzyme E2 variant 2					-2.058		
Unigene54677	protein polyubiquitination (GO:0000209)					1.751	2.123	

Unigene1912	protein deubiquitination (GO:0016579)					1.057		
Unigene10198	regulation of ubiquitin-protein ligase activity involved in mitotic cell cycle (GO:0051439)					-1.699		
Unigene19577	regulation of ubiquitin-protein ligase activity involved in mitotic cell cycle (GO:0051439)					-1.376		-1.015
Unigene53069	positive regulation of ubiquitin-protein ligase activity (GO:0051443)					-1.291		
Unigene31648	ubiquitin-dependent protein catabolic process (GO:0006511)					-1.247		-1.299
Unigene537	positive regulation of proteasomal ubiquitin-dependent protein catabolic process (GO:0032436)					-1.172		-1.324
Unigene53210	regulation of ubiquitin-protein ligase activity involved in mitotic cell cycle (GO:0051439)					-1.077		
Unigene8153	ubiquitin-protein ligase activity						3.123	
Unigene47307	ubiquitin carboxyl-terminal hydrolase 2/21						3.008	
Unigene33705	ubiquitin-protein ligase activity; Ubiquitin conjugation factor E4 B						2.352	
Unigene35169	E3 ubiquitin-protein ligase MYCBP2						2.095	
Unigene56677	ubiquitin carboxyl-terminal hydrolase 9/24						2.008	
Unigene9424	E3 ubiquitin-protein ligase RNF216						1.982	
Unigene43164	AN1-type zinc finger and ubiquitin domain-containing protein 1						1.934	
Unigene51664	E3 ubiquitin-protein ligase TRIP12						1.888	
Unigene37493	E3 ubiquitin-protein ligase HUWE1						1.856	
Unigene44705	E3 ubiquitin-protein ligase RNF149						1.845	
Unigene48840	ubiquitin carboxyl-terminal hydrolase 2/21						1.836	
Unigene49151	ubiquitin homeostasis (GO:0010992)						1.745	
Unigene15873	cullin-RING ubiquitin ligase complex; ubiquitin protein ligase binding						1.652	
Unigene53737	ubiquitin-protein ligase 1						1.541	
Unigene40767	E3 ubiquitin-protein ligase MYCBP2						1.352	
Unigene48412	ubiquitin-activating enzyme E1						1.326	
Unigene47510	E3 ubiquitin-protein ligase MYCBP2						1.276	
Unigene50240	E3 ubiquitin-protein ligase XIAP						1.232	
Unigene48078	E3 ubiquitin-protein ligase HERC4						1.230	
Unigene33407	ubiquitin-conjugating enzyme E2 R						1.182	
Unigene49433	E3 ubiquitin-protein ligase MYCBP2						1.163	
Unigene46690	ubiquitin carboxyl-terminal hydrolase 34						1.153	
Unigene39309	E3 ubiquitin-protein ligase RNF139						1.140	
Unigene20108	ubiquitin-conjugating enzyme E2 L3						1.127	
Unigene42456	ubiquitin carboxyl-terminal hydrolase 2/21						1.092	
Unigene9685	ubiquitin thioesterase CYLD						1.082	
Unigene48535	E3 ubiquitin-protein ligase MARCH6						1.059	
Unigene45917	ubiquitin carboxyl-terminal hydrolase 25/28						-1.221	
Unigene35181	E3 ubiquitin-protein ligase UBR4							3.051
Unigene19739	ubiquitin protein ligase binding (GO:0031625)							2.546
Unigene30584	ubiquitin thioesterase CYLD							1.320
Unigene53697	ubiquitin-conjugating enzyme E2 D/E							-1.565
CL4650.Contig1	polyubiquitin 5							-1.520

Unigene53182	ubiquitin							-1.448
Unigene9773	ubiquitin carboxyl-terminal hydrolase 6/32							-1.223
Unigene10075	ubiquitin-conjugating enzyme E2 C							-1.092
**4. Immunity**								
Unigene10443	immune system process (GO:0002376); response to stress (GO:0006950); positive regulation of apoptotic process (GO:0043065); apoptotic signaling pathway (GO:0097190)	1.318			1.104	1.248		
Unigene40466	salivary gland cell autophagic cell death	2.134						
Unigene10836	defensin B; defense response (GO:0006952)	1.486	1.357				-1.317	
Unigene28866	salivary gland cell autophagic cell death (GO:0035071); autophagy (GO:0006914)		-1.195				1.033	
CL1499.Contig2	Programmed cell death protein 7		-1.051					
Unigene52187	compound eye retinal cell programmed cell death (GO:0046667)		-1.034				1.109	
CL3176.Contig1	salivary gland cell autophagic cell death (GO:0035071)				2.98	2.895		
Unigene24223	compound eye retinal cell programmed cell death (GO:0046667)				-1.549	-3.273		
Unigene44279	positive regulation of immune response (GO:0050778)			1.074		1.009		
Unigene17517	ecdysone-mediated induction of salivary gland cell autophagic cell death (GO:0035072); activation of cysteine-type endopeptidase activity involved in apoptotic process (GO:0006919); regulation of gene silencing (GO:0060968)			2.14		2.084		
Unigene47423	negative regulation of apoptotic process (GO:0043066)			2.327		2.019	1.330	
Unigene46090	negative regulation of neuron apoptotic process (GO:0043524)			1.286		1.24	1.719	
Unigene39516	positive regulation of apoptotic process (GO:0043065)			1.192		1.388	1.867	
CL99.Contig1	apoptotic process (GO:0006915)			1.107				
Unigene49105	negative regulation of neuron apoptotic process (GO:0043524); response to heat (GO:0009408);			1.042	1.326	1.413		
CL574.Contig1	response to heat (GO:0009408); positive regulation of translational initiation (GO:0045948)				-1.78	-1.915		
CL2540.Contig1	defense response to Gram-negative bacterium (GO:0050829); activation of innate immune response (GO:0002218)					1.635		1.308
Unigene43286	mucosal immune response (GO:0002385); cellular response to osmotic stress (GO:0071470); response to salt stress (GO:0009651); cellular response to heat (GO:0034605); stress-activated MAPK cascade (GO:0051403)					1.487	1.758	1.148

Unigene51779	negative regulation of apoptotic process (GO:0043066); response to oxidative stress GO:0006979); adult chitin-based cuticle development GO:0008365); defense response to bacterium GO:0042742); mucosal immune response GO:0002385)					1.412		
Unigene22199	innate immune response (GO:0045087); regulation of apoptotic process (GO:0042981); positive regulation of compound eye retinal cell programmed cell death (GO:0046672)					1.061		
Unigene10853	immune response (GO:0006955); defense response (GO:0006952); Toll signaling pathway (GO:0008063)					1.039		
Unigene34974	innate immune response (GO:0045087); positive regulation of interferon-beta production (GO:0032728)					-2.455		
Unigene45716	Interferon, gamma-inducible protein 30; DNA-directed RNA polymerases I and III subunit RPAC1				1.257	1.848		
Unigene48198	Interferon, gamma-inducible protein 30; DNA-directed RNA polymerases I and III subunit RPAC1			1.811		1.826		
Unigene35309	Interferon, gamma-inducible protein 30; DNA-directed RNA polymerases I and III subunit RPAC1					1.888		
Unigene10338	Interferon, gamma-inducible protein 30; DNA-directed RNA polymerases I and III subunit RPAC1					1.219		
Unigene15628	positive regulation of adaptive immune response (GO:0002821); negative regulation of apoptotic process (GO:0043066); regulation of smooth muscle cell apoptotic process (GO:0034391); protein ubiquitination (GO:0016567); proteasomal ubiquitin-dependent protein catabolic process (GO:0043161)					-1.929		
Unigene9094	regulation of apoptotic process (GO:0042981); B cell mediated immunity (GO:0019724)					-1.871		
Unigene10598	regulation of immune response (GO:0050776)					-1.671		
Unigene9634	protein K11-linked ubiquitination (GO:0070979); apoptotic process (GO:0006915); innate immune response (GO:0045087)					-1.436		
Unigene46620	positive regulation of programmed cell death (GO:0043068)					2.508		
Unigene44782	salivary gland cell autophagic cell death (GO:0035071)					1.985		
Unigene46276	salivary gland cell autophagic cell death (GO:0035071)					1.895		
CL5350.Contig7	positive regulation of programmed cell death (GO:0043068)					1.434	1.675	
Unigene310	salivary gland cell autophagic cell death (GO:0035071)					1.352	1.272	
Unigene51842	negative regulation of cell death (GO:0060548)					1.067		1.03

Unigene49983	programmed cell death protein 8 (apoptosis-inducing factor)					1.053	1.187	
Unigene15708	positive regulation of compound eye retinal cell programmed cell death (GO:0046672)					-2.583		
Unigene20116	positive regulation of cell death (GO:0010942)					-1.764		-1.78
Unigene39207	positive regulation of motor neuron apoptotic process (GO:2000673)					2.18		
Unigene46228	apoptotic cell clearance (GO:0043277)					2.169	2.593	
Unigene24035	apoptotic process (GO:0006915); epithelial cell differentiation (GO:0030855)					1.653		1.363
Unigene14825	negative regulation of apoptotic process (GO:0043066)					1.566		
Unigene48158	negative regulation of apoptotic process (GO:0043066); cellular response to stress (GO:0033554)					1.248		
Unigene44630	positive regulation of intrinsic apoptotic signaling pathway (GO:2001244)					1.235		
Unigene45789	neuron apoptotic process (GO:0051402)					1.076		
Unigene33643	negative regulation of apoptotic process (GO:0043066)					1.071		
Unigene31625	apoptotic mitochondrial changes (GO:0008637); determination of adult lifespan (GO:0008340)					-3.455		
Unigene17634	apoptotic process (GO:0006915)					-2.975		
Unigene52616	apoptotic process (GO:0006915); autophagy (GO:0006914)					-2.112		
Unigene54953	viral reproduction (GO:0016032); negative regulation of ubiquitin-protein ligase activity involved in mitotic cell cycle (GO:0051436); positive regulation of ubiquitin-protein ligase activity involved in mitotic cell cycle (GO:0051437); negative regulation of programmed cell death (GO:0043069); regulation of apoptotic process (GO:0042981)					-1.949		
Unigene8502	apoptotic process (GO:0006915)					-1.894		-1.488
Unigene1020	cysteine-type endopeptidase activator activity involved in apoptotic process (GO:0008656); virus-host interaction (GO:0019048)					-1.778		-1.164
Unigene9204	negative regulation of apoptotic process (GO:0043066)					-1.772		
Unigene10190	protein polyubiquitination (GO:0000209); positive regulation of ubiquitin-protein ligase activity involved in mitotic cell cycle (GO:0051437); negative regulation of ubiquitin-protein ligase activity involved in mitotic cell cycle (GO:0051436); viral reproduction (GO:0016032);					-1.65		-1.248

Unigene21600	negative regulation of ubiquitin-protein ligase activity involved in mitotic cell cycle (GO:0051436); regulation of apoptotic process (GO:0042981); virus-host interaction (GO:0019048); positive regulation of ubiquitin-protein ligase activity involved in mitotic cell cycle (GO:0051437)					-1.497		
Unigene28552	negative regulation of apoptotic process (GO:0043066)					-1.46		-1.422
Unigene10215	positive regulation of ubiquitin-protein ligase activity involved in mitotic cell cycle (GO:0051437); regulation of apoptotic process (GO:0042981); viral reproduction (GO:0016032); negative regulation of ubiquitin-protein ligase activity involved in mitotic cell cycle (GO:0051436)					-1.455		
Unigene21757	protein polyubiquitination (GO:0000209); positive regulation of ubiquitin-protein ligase activity involved in mitotic cell cycle (GO:0051437); regulation of apoptotic process (GO:0042981); negative regulation of ubiquitin-protein ligase activity involved in mitotic cell cycle (GO:0051436); virus-host interaction (GO:0019048)					-1.455		
Unigene28926	protein polyubiquitination (GO:0000209); positive regulation of ubiquitin-protein ligase activity involved in mitotic cell cycle (GO:0051437); regulation of apoptotic process (GO:0042981); viral reproduction (GO:0016032); negative regulation of ubiquitin-protein ligase activity involved in mitotic cell cycle (GO:0051436)					-1.411		-1.222
Unigene52326	apoptotic process (GO:0006915)					-1.281		
CL4794.Contig1	negative regulation of apoptotic process (GO:0043066)					-1.135		-1.090
Unigene52732	protein K11-linked ubiquitination (GO:0070979); apoptotic process (GO:0006915); innate immune response; (GO:0045087); negative regulation of type I interferon production (GO:0032480)					-1.09		-1.173
Unigene29432	apoptotic process (GO:0006915)					-1.08		-1.203
Unigene9671	apoptotic process (GO:0006915); virus-host interaction (GO:0019048); autophagy (GO:0006914)					-1.059		
Unigene23095	negative regulation of apoptotic process (GO:0043066)					-1.045		-1.194
Unigene52714	negative regulation of apoptotic process (GO:0043066)					-1.033		
Unigene52865	T-complex protein 1 subunit epsilon; response to virus (GO:0009615)					-1.696		
Unigene47899	innate immune response (GO:0045087)						1.901	

Unigene46485	immune response-regulating cell surface receptor signaling pathway involved in phagocytosis (GO:0002433); regulation of defense response to virus by virus (GO:0050690); viral reproduction (GO:0016032)						1.182	
Unigene19893	innate immune response (GO:0045087); antibacterial humoral response (GO:0019731); antifungal humoral response (GO:0019732);defense response to Gram-negative bacterium (GO:0050829);defense response to protozoan (GO:0042832)						1.137	
Unigene50963	innate immune response (GO:0045087)						1.005	
Unigene8454	programmed cell death 6-interacting protein						2.671	
Unigene10914	salivary gland cell autophagic cell death (GO:0035071)						1.459	
Unigene46468	salivary gland cell autophagic cell death (GO:0035071)						1.363	
CL3176.Contig2	salivary gland cell autophagic cell death (GO:0035071)						1.232	
Unigene51152	autophagy-related protein 7						1.686	
Unigene20848	autophagy-related protein 2 homolog A						1.482	
Unigene48072	autophagy-related protein 9A						1.247	
Unigene9725	autophagy-related protein 101-like isoform 1						1.184	
Unigene51767	autophagy-related protein 13 homolog; autophagy (GO:0006914)						1.020	
Unigene53357	autophagy-related protein 4						-1.107	
Unigene50589	negative regulation of neuron apoptotic process (GO:0043524)						1.390	
Unigene19748	apoptotic cell clearance (GO:0043277)						1.353	
Unigene41971	negative regulation of neuron apoptotic process (GO:0043524)						1.259	
Unigene48727	TP53 apoptosis effector						1.251	
Unigene24181	negative regulation of apoptotic process (GO:0043066)						1.045	
Unigene46894	apoptotic process (GO:0006915)						1.002	
Unigene52764	response to interferon-gamma (GO:0034341)							-1.424
Unigene52336	cellular response to interferon-gamma (GO:0071346); neuron apoptotic process (GO:0051402)							-1.191
CL3398.Contig1	cellular response to interferon-gamma (GO:0071346)							-1.046
Unigene52240	autophagy-related protein 7							1.026
Unigene39244	autophagy (GO:0006914)							1.012
Unigene29924	ubiquitin-like protein Nedd8; ubiquitin protein ligase binding (GO:0031625); apoptotic process (GO:0006915)							-2.100
CL4655.Contig1	induction of apoptosis (GO:0006917)							-1.365
Unigene20857	engulfment of apoptotic cell (GO:0043652)							-1.352
Unigene52334	induction of apoptosis (GO:0006917); 40S ribosomal protein S3a							-1.145

Unigene42146	activation of cysteine-type endopeptidase activity involved in apoptotic process (GO:0006919); polyubiquitin binding (GO:0031593)							-1.063
**5. RNA interference**								
Unigene47276	mRNA 3'-UTR binding; RNA interference	1.142		1.839	1.562	1.705	1.311	
Unigene924	siRNA loading onto RISC involved in chromatin silencing by small RNA (GO:0070923); ubiquitin-protein ligase activity (GO:0004842)		-1.145			1.002	1.214	
Unigene21529	gene silencing (GO:0016458); regulation of transcription, DNA-dependent (GO:0006355); instar larval or pupal development (GO:0002165); wing disc development (GO:0035220)				-1.699	-2.224		
Unigene20470	Argonaute; gene silencing by miRNA (GO:0035195); eukaryotic translation initiation factor				-1.475	-2.371		
Unigene22628	negative regulation of viral genome replication (GO:0045071); production of siRNA involved in RNA interference (GO:0030422); gene silencing by miRNA (GO:0035195)					1.386	1.514	
Unigene25547	RNA interference (GO:0016246); instar larval development (GO:0002168); RNA-induced silencing complex (GO:0016442)					-2.571		
Unigene54734	RNA interference (GO:0016246); regulation of cell proliferation (GO:0042127); positive regulation of growth rate (GO:0040010);					-2.072		-1.869
Unigene19426	RNA interference (GO:0016246); instar larval development (GO:0002168)					-1.233		
Unigene16284	positive regulation of gene silencing by miRNA (GO:2000637)					1.782		
Unigene19426	RNA-induced silencing complex (GO:0016442); instar larval development (GO:0002168); RNA interference (GO:0016246)					-1.233		
Unigene38977	RNA-induced silencing complex (GO:0016442); pre-miRNA processing (GO:0031054);production of siRNA involved in RNA interference (GO:0030422)						2.178	
Unigene30738	positive regulation of gene silencing by miRNA (GO:2000637)						1.099	
Unigene49573	RNA-induced silencing complex (GO:0016442); RNA interference (GO:0016246)						1.083	
**6. Takeout protein**								
Unigene39342	protein takeout-like	3.75	3.045	3.538	4.092	3.92		
Unigene46756	protein takeout precursor	3.139	1.216	3.188	2.702	2.232		
Unigene47016	protein takeout precursor	2.743	1.102	2.857	2.132	2.427		
Unigene48270	protein takeout-like	1.315				1.232		
CL1088.contig1	protein takeout-like		1.304	1.47		1.116		
Unigene11525	protein takeout-like					1.955		
Unigene49724	protein takeout-like					1.084		

Unigene44854	takeout/JHBP like protein					-1.009		
**7. Cytochrome P450**								
Unigene52300	cytochrome P450	2.875	1.76	2.633	2.3	1.88		
Unigene49059	cytochrome P450	2.139		1.765		1.032	1.008	
Unigene21534	cytochrome P450		-1.441				1.473	
Unigene26550	cytochrome P450				4.357	4.152		
Unigene19603	cytochrome P450				1.797			
CL586.Contig2	cytochrome P450				1.03	1.162		
Unigene36040	cytochrome P450			2.937		2.451		
Unigene8880	cytochrome P450					2.451		
Unigene12253	cytochrome P450					2.089		
Unigene35427	cytochrome P450					1.589		
Unigene9226	cytochrome P450					1.514		
Unigene50523	cytochrome P450					1.275		
Unigene49336	cytochrome P450					1.173		
Unigene47397	cytochrome P450					1.078	1.587	
CL586.Contig1	cytochrome P450						1.283	
Unigene9290	cytochrome P450							-1.289
**8. Chemosensory protein**								
Unigene95	chemosensory protein; response to virus	2.712		2.35	2.453	2.597		
Unigene34638	chemosensory protein CSP4; response to virus	1.135		1.402			1.538	
Unigene32037	putative chemosensory protein CSP8		1.821		1.486	2.039		
Unigene44196	putative chemosensory protein CSP9				1.382	1.667	1.166	
Unigene48970	chemosensory protein			1.19	1.256	1.298		
Unigene32978	chemosensory protein; response to virus (GO:0009615)					-1.031		
Unigene56754	putative chemosensory protein CSP3						1.682	
Unigene37785	putative chemosensory protein CSP8							-2.245
**9. Heat shock pathway components**								
Unigene47833	putative small heat shock protein	1.338			4.237	4.863		
Unigene20655	heat shock protein 70 (Hsp70), putative		1.527					
Unigene9991	Hsp20 domain-containing protein		1.085		1.583			-1.228
Unigene19103	putative small heat shock protein				7.85	8.397		
CL954.Contig1	heat shock protein 70				7.515	8.229		
Unigene43012	putative small heat shock protein				7.435	7.8		
CL3715.Contig1	heat shock protein 70kDa				7.075	7.78		
Unigene18906	heat shock protein 68				6.818	7.746		
Unigene5646	heat shock protein 70				6.274	7.185		
Unigene8341	heat shock protein 78				3.886	3.169		
Unigene18977	heat shock protein 101				3.833	2.854		
Unigene12447	heat shock protein 101				3.814	2.997		
Unigene29561	heat shock protein 78				2.502			
Unigene730	heat shock protein 90				1.753	1.854		
Unigene52268	heat shock protein 70kDa				1.159			-1.136
Unigene9618	heat shock 70 kDa protein 4L isoform 1				1.142	1.789	1.02	
Unigene46914	heat shock protein 40				1.114	1.843		
Unigene34043	Heat-shock protein 110 kDa				1.019	1.062		
Unigene52873	heat shock transcription factor (Hsf1 protein); response to stress (GO:0006950)				-1.038	-2.455		-1.418

Unigene16177	heat shock 70kDa protein					-2.299		-1.541
Unigene9952	heat shock 70kDa protein					-1.597		-1.134
Unigene9697	heat shock 70kDa protein; response to heat (GO:0009408)					-1.267		-1.019
Unigene10649	heat shock 70kDa protein; ubiquitin protein ligase binding (GO:0031625); cysteine-type endopeptidase inhibitor activity involved in apoptotic process (GO:0043027)					-1.055		-1.028
Unigene30980	heat shock 70kDa protein					-1.019		-1.097
Unigene52319	heat shock 70kDa protein; response to heat (GO:0009408); negative regulation of apoptotic process (GO:0043066)					-1.001		-1.151
Unigene49244	Heat shock 70 kDa protein cognate, putative; response to heat (GO:0009408)					1.141		
Unigene51830	heat shock protein 70 HSP70 interacting protein, putative; response to stress (GO:0006950)					1.121		
Unigene47562	Activator of 90 kDa heat shock protein ATPase-like protein 1					1.072		
Unigene52266	10 kDa heat shock protein, mitochondrial-like					-1.118		
Unigene52263	heat shock protein 90a; response to stress (GO:0006950)							-1.229
Unigene10095	stress-70 protein, mitochondrial-like; heat shock protein binding (GO:0031072)							-1.118
Unigene52373	Hsp70 protein binding (GO:0030544); stress-induced-phosphoprotein 1; regulation of JAK-STAT cascade (GO:0046425)					-1.088		-1.218
**10. Trehalose**								
Unigene47369	facilitated trehalose transporter		2.288				-2.019	
Unigene39864	facilitated trehalose transporter Tret1-like		-1.987				1.445	
Unigene48083	facilitated trehalose transporter		-1.516				1.764	
Unigene40046	trehalose 6-phosphate synthase		-1.384				1.308	
Unigene50244	facilitated trehalose transporter				1.409	2.015		
Unigene52848	facilitated trehalose transporter				-1.03			
Unigene50659	trehalose-6-phosphate synthase			2.847		2.814	2.330	
Unigene14073	facilitated trehalose transporter			1.834			1.724	
Unigene35193	facilitated trehalose transporter					1.425		
Unigene15721	facilitated trehalose transporter					1.201		1.071
Unigene33711	trehalose 6-phosphate synthase					-2.381		
Unigene33712	trehalose 6-phosphate synthase					-2.192		-1.672
Unigene53240	facilitated trehalose transporter						3.123	
CL5228.Contig2	facilitated trehalose transporter; positive regulation of JAK-STAT cascade (GO:0046427)						1.934	
**11. Apolipoprotein**								
CL3560.Contig2	Apolipoprotein D precursor, putative		-1.156					
CL3560.Contig1	Apolipoprotein D precursor, putative		-1.088					
Unigene55864	apolipoprotein D precursor			2.73		2.099	2.852	
CL2103	Apolipoprotein D precursor			1.633		1.623		
Unigene10970	apolipoprotein D-like			1.174				
Unigene18485	Apolipoprotein D					1.297		

CL2103.Contig2	Apolipoprotein D precursor						1.556	
Unigene25383	apolipoprotein D-like						1.083	

Supplementary Table S2: Enriched GO terms and differentially expressed Unigenes

GO term	List of related Unigenes	Number
**V5 relative to N5 (283 different Unigenes)**		
actin filament organization	Unigene51889, Unigene18457, Unigene44970, CL4555.Contig2, Unigene36722, CL3282.Contig1, Unigene22778, Unigene42205, CL2157.Contig2, Unigene51480, Unigene49545, Unigene28866, Unigene1017, Unigene52600, Unigene52187, Unigene23515, Unigene50661, Unigene1489, Unigene44969, Unigene38449, Unigene50663, Unigene19748, Unigene38448, Unigene44971, Unigene46485, Unigene8454	**26**
actin cytoskeleton organization	Unigene18457, Unigene49458, Unigene36722, Unigene22778, Unigene28866, Unigene14659, Unigene52600, Unigene49128, Unigene46758, Unigene48874, Unigene23515, Unigene24181, Unigene44969, Unigene51250, Unigene19748, Unigene50814, Unigene44971, Unigene50623, Unigene48954, Unigene46485, Unigene51889, CL4555.Contig2, Unigene44970, CL3282.Contig1, CL2157.Contig2, Unigene42205, Unigene49545, Unigene51480, CL677.Contig1, CL4144.Contig1, Unigene1017, CL110.Contig1, Unigene23513, Unigene52187, Unigene1489, Unigene50661, Unigene38449, Unigene50663, Unigene38448, Unigene8454	**40**
actin filament-based process	Unigene18457, Unigene49458, Unigene36722, Unigene22778, Unigene28866, Unigene14659, Unigene52600, Unigene49128, Unigene46758, Unigene48747, Unigene48874, Unigene23515, Unigene24181, Unigene44969, Unigene51250, Unigene19748, Unigene50814, Unigene44971, Unigene50623, Unigene48954, Unigene46485, Unigene51889, CL4555.Contig2, Unigene44970, CL3282.Contig1, CL2157.Contig2, Unigene42205, Unigene49545, Unigene51480, CL677.Contig1, CL4144.Contig1, Unigene1017, CL110.Contig1, Unigene23513, Unigene52187, Unigene1489, Unigene50661, Unigene38449, Unigene50663, Unigene38448, Unigene8454	**41**
actin filament bundle assembly	Unigene51889, Unigene18457, CL3282.Contig1, Unigene50661, Unigene28866, Unigene19748, Unigene50663, Unigene1017, Unigene46485	**9**
chaeta development	Unigene34041, CL3282.Contig1, Unigene42205, Unigene28866, Unigene1017, Unigene42812, Unigene46758, Unigene48747, Unigene50292, Unigene51617, Unigene41354, Unigene52187, Unigene47423, Unigene38449, Unigene51618, Unigene47977, Unigene38448	**17**
protein complex subunit organization	Unigene18457, Unigene49151, CL3176.Contig2, Unigene48029, Unigene36722, Unigene22778, Unigene9879, Unigene28866, Unigene52600, Unigene49701, Unigene32956, Unigene48874, Unigene23515, Unigene47423, Unigene44969, Unigene19748, Unigene46893, Unigene48865, Unigene44971, Unigene48954, Unigene46485, CL1207.Contig3, Unigene51889, CL4555.Contig2, Unigene44970, CL3282.Contig1, CL2157.Contig2, Unigene42205, Unigene49545, Unigene50216, Unigene51480, Unigene51380, CL4144.Contig1, CL5044.Contig1, Unigene1017, Unigene51696, Unigene1215, CL110.Contig1, Unigene52187, Unigene50661, Unigene1489, Unigene50214, Unigene38449, Unigene50663, Unigene46894, Unigene38448, Unigene51692, Unigene32955, CL4570.Contig1, Unigene8454	**50**
cell adhesion	Unigene51817, Unigene18457, Unigene49178, Unigene33023, Unigene22778, Unigene10914, CL2157.Contig2, Unigene55242, Unigene51480, Unigene47164, Unigene35923, Unigene49701, Unigene49177, Unigene49128, Unigene46258, Unigene21802, Unigene51674, CL1434.Contig2, Unigene49868, Unigene48874, CL3602.Contig2, Unigene52187, Unigene50661, Unigene47423, Unigene1489, Unigene45802, Unigene46513, Unigene50663, Unigene19748, Unigene49268, Unigene45437, Unigene49808, Unigene46485	**33**

biological adhesion	Unigene51817, Unigene18457, Unigene49178, Unigene33023, Unigene22778, Unigene10914, CL2157.Contig2, Unigene55242, Unigene51480, Unigene47164, Unigene35923, Unigene49701, Unigene49177, Unigene49128, Unigene46258, Unigene21802, Unigene51674, CL1434.Contig2, Unigene49868, Unigene48874, CL3602.Contig2, Unigene52187, Unigene50661, Unigene47423, Unigene1489, Unigene45802, Unigene46513, Unigene50663, Unigene19748, Unigene49268, Unigene45437, Unigene49808, Unigene46485	**33**
extracellular region	Unigene9745, Unigene49151, Unigene41819, Unigene47072, Unigene41899, Unigene50608, Unigene49324, CL1402.Contig3, Unigene47070, CL2895.Contig3, Unigene34638, Unigene51395, Unigene55864, Unigene23515, CL2103.Contig1, Unigene44969, CL5351.Contig1, Unigene48783, Unigene19893, Unigene52987, CL5285.Contig1, Unigene44971, Unigene1661, Unigene49115, Unigene44970, Unigene534, Unigene51958, Unigene52125, Unigene18030, CL2103.Contig2, Unigene334, Unigene51850, CL5285.Contig11, Unigene43001, Unigene33919, Unigene51599, Unigene51541, Unigene35035, Unigene185	**39**
lipid particle	Unigene1304, Unigene35623, Unigene22778, CL2157.Contig2, Unigene51480, CL1994.Contig2, CL5044.Contig1, Unigene49573, Unigene46093, Unigene25073, Unigene53878, Unigene32956, Unigene31760, Unigene47423, Unigene47625, CL5046.Contig1, Unigene49268, CL1703.Contig2, Unigene47977, Unigene31433, Unigene32955, CL401.Contig4	**22**
membrane	Unigene18457, Unigene49151, Unigene48223, Unigene35105, Unigene46231, Unigene44825, Unigene41463, Unigene48805, Unigene43148, CL2786.Contig1, Unigene49642, Unigene35919, Unigene42429, Unigene1582, Unigene18220, Unigene30738, Unigene39376, Unigene45087, Unigene23914, Unigene51938, Unigene51817, Unigene23437, Unigene534, Unigene51480, Unigene43760, Unigene33140, Unigene18795, Unigene49845, Unigene45792, Unigene42093, Unigene41354, Unigene49567, Unigene51978, Unigene49868, Unigene19582, Unigene50701, Unigene1296, Unigene47476, Unigene1489, Unigene50663, Unigene30278, Unigene45437, Unigene48727, Unigene45747, Unigene42809, Unigene45822, Unigene37786, CL4224.Contig2, Unigene46638, Unigene22778, Unigene9879, Unigene40262, Unigene49701, Unigene43762, Unigene49682, Unigene36160, CL4452.Contig1, Unigene50689, Unigene21802, Unigene42295, Unigene59854, Unigene52402, Unigene47423, Unigene35055, Unigene34716, CL3193.Contig1, Unigene33924, Unigene57316, Unigene19281, Unigene49808, Unigene48142, Unigene54023, Unigene46485, Unigene26882, CL1207.Contig3, Unigene33084, Unigene44321, Unigene49571, Unigene34428, Unigene19165, Unigene44148, Unigene50993, Unigene53240, CL723.Contig2, CL1200.Contig1, Unigene49783, Unigene33524, Unigene42672, Unigene50661, CL4125.Contig3, Unigene46693, Unigene54933, Unigene41592, Unigene46162, Unigene45165, Unigene22461, Unigene49411, Unigene45494, Unigene51178, Unigene743, Unigene43286, Unigene8316, CL689.Contig2, Unigene37347, Unigene35923, Unigene39978, Unigene21542, Unigene10664, Unigene51617, Unigene42267, Unigene46496, Unigene50571, Unigene45802, Unigene51603, CL2815.Contig2, Unigene50825, Unigene45637, Unigene44826, Unigene47164, Unigene53349, Unigene47806, Unigene21864, CL906.Contig2, Unigene46258, Unigene46863, Unigene39997, Unigene50414, Unigene51765, Unigene54074, Unigene36724, Unigene45168, Unigene51511, CL3193.Contig2, Unigene1821, Unigene40708, Unigene1669, Unigene43776, Unigene48029, Unigene44322, Unigene47904, Unigene55674, Unigene21156, Unigene52600, Unigene49177, Unigene46498, Unigene21751, Unigene51674, Unigene49674, Unigene39516, Unigene49979, Unigene24181, Unigene42296, CL4332.Contig2, Unigene47899, Unigene44659, Unigene20107, Unigene34670, Unigene49178, Unigene51676, Unigene33023, Unigene10914, CL2157.Contig2, Unigene46701, Unigene49978, Unigene1017, Unigene50343, Unigene1215, Unigene42268,	**175**
actin binding	Unigene21806, Unigene51889, Unigene44970, Unigene43174, Unigene36722, CL3282.Contig1, Unigene22778, Unigene42205, CL2157.Contig2, Unigene55242, Unigene51480, CL677.Contig1, Unigene14659, Unigene33138, Unigene43148, Unigene48747, CL110.Contig1, Unigene39331, Unigene48868, Unigene43413, Unigene23515, Unigene44969, Unigene38449, CL110.Contig2, Unigene46513, Unigene38448, Unigene23727, Unigene48170, Unigene44971	**29**
cytoskeletal protein binding	Unigene45494, Unigene43174, Unigene36722, Unigene22778, Unigene55944, Unigene14659, Unigene43148, Unigene42526, Unigene48747, Unigene41971, Unigene32956, Unigene46690, Unigene23515, Unigene44969, Unigene48170, Unigene44971, Unigene21806, Unigene51889, Unigene44970, CL3282.Contig1, CL2157.Contig2, Unigene42205, Unigene51480, Unigene55242, CL677.Contig1, Unigene33138, Unigene52916, CL110.Contig1, Unigene39331, Unigene48868, Unigene43413, Unigene51011, Unigene38449, CL110.Contig2, Unigene46513, Unigene38448, Unigene23727, Unigene32955	**38**
**V36 relative to N36 (291 different Unigenes)**		

viral genome expression	CL990.Contig2, Unigene52332, CL910.Contig2, Unigene10195, Unigene9620, Unigene52447, Unigene30706, Unigene52590, CL2431.Contig3, Unigene52338	**10**
viral transcription	CL990.Contig2, Unigene52332, CL910.Contig2, Unigene10195, Unigene9620, Unigene52447, Unigene30706, Unigene52590, CL2431.Contig3, Unigene52338	**10**
viral reproduction	Unigene10190, CL990.Contig2, Unigene11676, Unigene52332, CL910.Contig2, Unigene28926, Unigene10195, Unigene9620, Unigene30706, Unigene52447, Unigene52590, CL2431.Contig3, CL2943.Contig2, Unigene1020, Unigene52338	**15**
multi-organism cellular process	Unigene10190, CL990.Contig2, Unigene11676, Unigene52332, CL910.Contig2, Unigene28926, Unigene10195, Unigene9620, Unigene30706, Unigene52447, Unigene52590, CL2431.Contig3, CL2943.Contig2, Unigene1020, Unigene52338	**15**
protein targeting to ER	CL990.Contig2, Unigene52332, CL910.Contig2, Unigene52764, Unigene10195, Unigene9620, Unigene52447, Unigene30706, Unigene52590, CL2431.Contig3, Unigene52338	**11**
establishment of protein localization to endoplasmic	CL990.Contig2, Unigene52332, CL910.Contig2, Unigene52764, Unigene10195, Unigene9620, Unigene52447, Unigene30706, Unigene52590, CL2431.Contig3, Unigene52338	**11**
translational termination	CL990.Contig2, Unigene52332, CL910.Contig2, Unigene10195, Unigene9620, Unigene52447, Unigene30706, Unigene52590, CL2431.Contig3, Unigene52338	**10**
viral infectious cycle	CL990.Contig2, Unigene52332, CL910.Contig2, Unigene10195, Unigene9620, Unigene52447, Unigene30706, Unigene52590, CL2431.Contig3, Unigene52338	**10**
protein localization to endoplasmic reticulum	CL990.Contig2, Unigene52332, CL910.Contig2, Unigene52764, Unigene10195, Unigene9620, Unigene52447, Unigene30706, Unigene52590, CL2431.Contig3, Unigene52338	**11**
cellular metabolic process	Unigene10079, CL990.Contig2, Unigene33006, Unigene52324, CL5309.Contig1, Unigene8259, Unigene30706, Unigene52810, Unigene52732, CL5033.Contig2, Unigene39521, Unigene33552, CL4794.Contig1, Unigene19978, Unigene9670, Unigene23422, Unigene51321, Unigene27336, Unigene25581, Unigene10378, Unigene52644, Unigene29924, Unigene30135, Unigene38849, Unigene9290, Unigene22035, Unigene52562, Unigene33439, Unigene42147, Unigene10034, Unigene9687, Unigene52926, Unigene9179, CL3398.Contig1, Unigene11495, Unigene10751, CL112.Contig1, Unigene10041, Unigene52769, Unigene31292, Unigene8404, Unigene52590, Unigene16063, Unigene54755, Unigene473, Unigene29233, Unigene24445, Unigene20593, Unigene22778, Unigene33331, Unigene9677, Unigene53320, Unigene99, Unigene53895, Unigene28586, CL2267.Contig2, Unigene16133, Unigene53100, Unigene29477, Unigene52381, CL167.Contig2, Unigene10654, Unigene10312, Unigene28926, Unigene52667, Unigene10007, CL240.Contig1, Unigene33259, Unigene30918, Unigene16177, Unigene46715, Unigene9627, Unigene33606, Unigene52332, Unigene32069, Unigene20206, Unigene34417, Unigene43948, Unigene10226, Unigene33311, Unigene11676, Unigene11765, Unigene52649, Unigene9952, Unigene19323, Unigene46198, Unigene33507, Unigene52279, CL3235.Contig1, Unigene10649, Unigene1020, Unigene52838, Unigene52327, Unigene33668, Unigene30808, Unigene28660, Unigene9001, Unigene12267, Unigene52328, Unigene11530, Unigene32952, Unigene22431, Unigene537, Unigene54734, Unigene10541, CL359.Contig1, Unigene8272, Unigene9775, Unigene53958, Unigene52323, Unigene10694, Unigene32831, Unigene52289, Unigene10008, CL2431.Contig3, Unigene19560, CL4159.Contig2, Unigene52338, Unigene52524, Unigene19739, Unigene19577, Unigene10095, Unigene44323, Unigene52263, Unigene52614, Unigene53503, CL718.Contig1, Unigene40246, Unigene45582, Unigene46064, Unigene23918, Unigene42146, CL3841.Contig1, Unigene10060, Unigene52808, CL2943.Contig2, Unigene32921, CL4655.Contig1, Unigene55020, Unigene52179, Unigene9917, Unigene31648, CL3555.Contig2, Unigene9620, Unigene15125, Unigene39276, Unigene10973, Unigene52313, Unigene52557, Unigene52636, Unigene39931, Unigene53050, Unigene53878, Unigene16329, Unigene18792, Unigene39553, Unigene52334, CL910.Contig2, Unigene10261, Unigene10195, Unigene30673, Unigene52447, Unigene34361, Unigene30980, Unigene33870, Unigene52515, Unigene29212, Unigene52905, Unigene32094, Unigene48898, Unigene38188, Unigene53860, CL3725.Contig2, Unigene52336, Unigene10146, Unigene52409, Unigene52768, Unigene28552, Unigene29431, Unigene49671, Unigene10190, Unigene46682, Unigene53034, CL3845.Contig2, Unigene29432, CL5144.Contig2	**188**

translation	Unigene10079, CL990.Contig2, Unigene52324, Unigene9620, Unigene30706, Unigene15125, Unigene52328, Unigene52313, CL4794.Contig1, Unigene52334, CL910.Contig2, Unigene52323, Unigene10195, Unigene10694, Unigene30673, Unigene52447, CL2431.Contig3, Unigene29212, Unigene52338, CL4159.Contig2, Unigene9627, Unigene30135, Unigene52332, Unigene22035, Unigene32069, Unigene52562, Unigene52614, Unigene10146, Unigene20206, Unigene53503, Unigene52409, Unigene52590, CL4655.Contig1, CL5144.Contig2, Unigene10025	**35**
SRP-dependent cotranslational protein targeting to membrane	CL990.Contig2, Unigene52332, CL910.Contig2, Unigene10195, Unigene9620, Unigene52447, Unigene30706, Unigene52590, CL2431.Contig3, Unigene52338	**10**
translational elongation	CL990.Contig2, Unigene52332, CL910.Contig2, Unigene52562, Unigene10195, Unigene30673, Unigene52614, Unigene9620, Unigene52447, Unigene30706, Unigene52590, CL2431.Contig3, CL5144.Contig2, Unigene52338, CL4794.Contig1	**15**
cotranslational protein targeting to membrane	CL990.Contig2, Unigene52332, CL910.Contig2, Unigene10195, Unigene9620, Unigene52447, Unigene30706, Unigene52590, CL2431.Contig3, Unigene52338	**10**
protein targeting to membrane	CL990.Contig2, Unigene52332, CL910.Contig2, Unigene10195, Unigene9620, Unigene52447, Unigene30706, Unigene52590, CL2431.Contig3, Unigene52338	**10**
establishment of protein localization to organelle	CL990.Contig2, Unigene23670, Unigene10095, Unigene52332, CL910.Contig2, Unigene52764, Unigene10195, Unigene9620, Unigene52447, Unigene30706, Unigene52590, CL2431.Contig3, Unigene52338	**13**
viral reproductive process	CL990.Contig2, Unigene52332, CL910.Contig2, Unigene10195, Unigene9620, Unigene52447, Unigene30706, Unigene52590, CL2431.Contig3, Unigene1020, Unigene52338	**11**
cellular protein complex disassembly	CL990.Contig2, Unigene52332, CL910.Contig2, Unigene10195, Unigene9620, Unigene52447, Unigene30706, Unigene52590, CL2431.Contig3, Unigene52338	**10**
protein complex disassembly	CL990.Contig2, Unigene52332, CL910.Contig2, Unigene10195, Unigene9620, Unigene52447, Unigene30706, Unigene52590, CL2431.Contig3, Unigene52338	**10**

metabolic process	Unigene10079, CL990.Contig2, Unigene33006, Unigene52324, CL5309.Contig1, Unigene8259, Unigene30706, Unigene52810, Unigene52732, CL5033.Contig2, Unigene39521, Unigene33552, CL4794.Contig1, Unigene19978, Unigene9670, Unigene23422, Unigene46761, Unigene51321, Unigene51842, Unigene27336, Unigene10879, Unigene25581, Unigene10378, Unigene52644, Unigene29924, Unigene30135, Unigene38849, Unigene9290, Unigene22035, Unigene52562, Unigene33439, Unigene42147, Unigene10034, Unigene9687, Unigene52926, Unigene9179, CL3398.Contig1, Unigene11495, Unigene10751, CL112.Contig1, Unigene10041, CL110.Contig2, Unigene52769, Unigene31292, Unigene8404, Unigene52590, Unigene39381, Unigene16063, Unigene54755, Unigene473, Unigene42119, Unigene29233, Unigene24445, Unigene20593, Unigene22778, Unigene33331, CL3376.Contig1, Unigene9677, Unigene53320, Unigene99, Unigene53617, Unigene53895, Unigene28586, CL2267.Contig2, Unigene16133, Unigene52435, Unigene53100, Unigene29477, Unigene52381, CL167.Contig2, Unigene10654, Unigene10312, Unigene28926, Unigene52667, Unigene10007, Unigene9084, Unigene52592, CL240.Contig1, Unigene33259, Unigene30918, Unigene20116, Unigene16177, Unigene46715, Unigene9627, Unigene33606, Unigene52332, Unigene32069, Unigene20206, Unigene52716, Unigene34417, Unigene43948, Unigene10226, Unigene33311, Unigene11676, Unigene11765, Unigene52649, Unigene9952, Unigene19323, Unigene24072, Unigene46198, Unigene33507, Unigene52279, CL3235.Contig1, Unigene10649, Unigene1020, Unigene52838, Unigene52327, Unigene33668, Unigene10743, Unigene52253, Unigene30808, Unigene28660, Unigene9001, Unigene12267, Unigene52328, Unigene11530, Unigene32952, Unigene537, Unigene22431, Unigene54734, Unigene10541, CL359.Contig1, Unigene8272, Unigene9775, Unigene51726, Unigene53958, Unigene52323, Unigene10694, Unigene32831, Unigene52289, Unigene10008, CL2431.Contig3, Unigene19560, CL4159.Contig2, Unigene52338, Unigene52524, Unigene19739, Unigene19577, Unigene10095, Unigene44323, Unigene52263, Unigene52614, Unigene53503, CL718.Contig1, Unigene40246, Unigene45582, Unigene46064, Unigene23918, Unigene42146, CL3841.Contig1, Unigene10060, Unigene52808, Unigene23095, Unigene45642, CL2943.Contig2, Unigene32921, Unigene10288, CL4655.Contig1, Unigene52179, Unigene55020, Unigene9917, Unigene20876, Unigene31648, Unigene19435, CL3555.Contig2, Unigene9620, Unigene15125, Unigene39276, Unigene10973, Unigene52313, Unigene52557, Unigene53050, Unigene52636, Unigene39931, Unigene53878, Unigene18792, Unigene16329, Unigene39553, Unigene52334, CL910.Contig2, Unigene10261, Unigene10195, Unigene30673, Unigene52447, Unigene34361, Unigene30980, Unigene33870, Unigene52515, Unigene29212, Unigene52905, Unigene32094, Unigene48898, Unigene38188, Unigene33712, Unigene53860, CL3725.Contig2, Unigene52336, Unigene48738, Unigene10557, Unigene10146, Unigene52409, Unigene52768, Unigene28552, Unigene29431, Unigene42992, Unigene49671, Unigene10190, Unigene46682, Unigene53034, CL3845.Contig2	**214**
organic substance metabolic process	Unigene10079, CL990.Contig2, Unigene33006, Unigene52324, Unigene8259, Unigene30706, Unigene52810, Unigene52732, CL5033.Contig2, Unigene39521, Unigene33552, CL4794.Contig1, Unigene19978, Unigene9670, Unigene23422, Unigene46761, Unigene51321, Unigene51842, Unigene27336, Unigene10879, Unigene25581, Unigene52644, Unigene29924, Unigene30135, Unigene38849, Unigene22035, Unigene52562, Unigene33439, Unigene42147, Unigene9687, Unigene52926, Unigene9179, CL3398.Contig1, Unigene11495, Unigene10751, CL112.Contig1, Unigene10041, Unigene52769, Unigene31292, Unigene8404, Unigene52590, Unigene16063, Unigene54755, Unigene473, Unigene29233, Unigene24445, Unigene20593, Unigene22778, Unigene33331, Unigene53320, Unigene99, Unigene53617, Unigene53895, Unigene28586, CL2267.Contig2, Unigene16133, Unigene53100, Unigene52381, CL167.Contig2, Unigene10654, Unigene28926, Unigene52667, Unigene10007, Unigene52592, CL240.Contig1, Unigene33259, Unigene30918, Unigene20116, Unigene16177, Unigene46715, Unigene9627, Unigene33606, Unigene52332, Unigene32069, Unigene20206, Unigene34417, Unigene43948, Unigene33311, Unigene11676, Unigene11765, Unigene52649, Unigene9952, Unigene19323, Unigene46198, Unigene33507, Unigene52279, CL3235.Contig1, Unigene10649, Unigene1020, Unigene52838, Unigene52327, Unigene33668, Unigene10743, Unigene52253, Unigene30808, Unigene28660, Unigene9001, Unigene12267, Unigene52328, Unigene11530, Unigene22431, Unigene537, Unigene54734, Unigene10541, CL359.Contig1, Unigene8272, Unigene9775, Unigene53958, Unigene52323, Unigene10694, Unigene32831, Unigene52289, Unigene10008, CL2431.Contig3, Unigene19560, CL4159.Contig2, Unigene52338, Unigene52524, Unigene19739, Unigene19577, Unigene10095, Unigene44323, Unigene52263, Unigene52614, Unigene53503, Unigene40246, Unigene46064, Unigene23918, Unigene42146, CL3841.Contig1, Unigene10060, Unigene52808, CL2943.Contig2, Unigene32921, Unigene10288, CL4655.Contig1, Unigene55020, Unigene52179, Unigene9917, Unigene20876, Unigene31648, CL3555.Contig2, Unigene9620, Unigene15125, Unigene39276, Unigene10973, Unigene52313, Unigene52557, Unigene52636, Unigene39931, Unigene53050, Unigene53878, Unigene16329, Unigene18792, Unigene39553, Unigene52334, CL910.Contig2, Unigene10261, Unigene10195, Unigene30673, Unigene52447, Unigene34361, Unigene30980, Unigene33870, Unigene52515, Unigene29212, Unigene52905, Unigene32094, Unigene48898, Unigene38188, CL3725.Contig2, Unigene52336, Unigene48738, Unigene10146, Unigene52409, Unigene52768, Unigene28552, Unigene29431, Unigene49671, Unigene10190	**187**

carboxylic acid metabolic process	Unigene24445, Unigene30808, Unigene33006, Unigene15125, Unigene39276, Unigene99, Unigene39521, Unigene22431, Unigene19978, Unigene53878, Unigene52381, Unigene23422, CL167.Contig2, Unigene39553, Unigene32831, Unigene34361, Unigene27336, Unigene25581, Unigene52289, Unigene30918, Unigene33606, Unigene32094, Unigene38849, Unigene22035, Unigene53503, Unigene40246, Unigene49671, Unigene52649, CL3398.Contig1, Unigene19323, Unigene23918, Unigene52769, Unigene52279, CL3845.Contig2, Unigene54755	**35**
organic substance biosynthetic process	Unigene10079, CL990.Contig2, Unigene30808, Unigene52324, Unigene30706, Unigene52328, Unigene52732, Unigene39521, Unigene22431, Unigene10541, CL4794.Contig1, CL359.Contig1, Unigene51321, Unigene52323, Unigene10694, Unigene32831, Unigene27336, Unigene25581, Unigene52289, CL2431.Contig3, Unigene52338, CL4159.Contig2, Unigene52644, Unigene30135, Unigene22035, Unigene52562, Unigene44323, Unigene52614, Unigene53503, Unigene40246, CL3398.Contig1, Unigene23918, Unigene42146, Unigene52769, Unigene52590, CL2943.Contig2, Unigene32921, Unigene54755, CL4655.Contig1, Unigene52179, Unigene29233, Unigene24445, Unigene33331, Unigene9620, Unigene15125, Unigene99, Unigene10973, Unigene28586, Unigene52313, Unigene52636, Unigene53100, Unigene16329, Unigene52334, CL910.Contig2, Unigene10195, Unigene30673, Unigene52447, Unigene34361, CL240.Contig1, Unigene33259, Unigene30918, Unigene33870, Unigene29212, Unigene20116, Unigene33606, Unigene9627, Unigene52336, Unigene52332, Unigene32069, Unigene10146, Unigene20206, Unigene34417, Unigene52409, Unigene49671, Unigene11676, Unigene11765, Unigene9952, Unigene52279, CL5144.Contig2, Unigene10025	**80**
macromolecular complex disassembly	CL990.Contig2, Unigene52332, CL910.Contig2, Unigene10195, Unigene9620, Unigene52447, Unigene30706, Unigene52590, CL2431.Contig3, Unigene52338	**10**
nuclear-transcribed mRNA catabolic process, nonsense-mediated decay	CL990.Contig2, Unigene52332, CL910.Contig2, Unigene10195, Unigene9620, Unigene52447, Unigene30706, Unigene52590, CL2431.Contig3, Unigene52338	**10**
primary metabolic process	Unigene10079, CL990.Contig2, Unigene52324, Unigene8259, Unigene30706, Unigene52810, Unigene52732, CL5033.Contig2, Unigene33552, CL4794.Contig1, Unigene19978, Unigene9670, Unigene23422, Unigene46761, Unigene51321, Unigene51842, Unigene27336, Unigene10879, Unigene25581, Unigene52644, Unigene29924, Unigene30135, Unigene38849, Unigene22035, Unigene52562, Unigene33439, Unigene42147, Unigene9687, Unigene52926, Unigene9179, CL3398.Contig1, Unigene11495, Unigene10751, CL112.Contig1, Unigene10041, Unigene52769, Unigene31292, Unigene8404, Unigene52590, Unigene16063, Unigene54755, Unigene29233, Unigene24445, Unigene20593, Unigene22778, Unigene33331, Unigene9677, Unigene53320, Unigene99, Unigene53617, Unigene28586, CL2267.Contig2, Unigene16133, Unigene53100, Unigene52381, CL167.Contig2, Unigene10654, Unigene10312, Unigene28926, Unigene52667, Unigene10007, Unigene52592, CL240.Contig1, Unigene33259, Unigene30918, Unigene20116, Unigene16177, Unigene46715, Unigene9627, Unigene33606, Unigene52332, Unigene32069, Unigene20206, Unigene34417, Unigene43948, Unigene33311, Unigene11676, Unigene11765, Unigene52649, Unigene19323, Unigene46198, Unigene33507, Unigene52279, CL3235.Contig1, Unigene10649, Unigene1020, Unigene52838, Unigene10743, Unigene33668, Unigene30808, Unigene28660, Unigene9001, Unigene12267, Unigene52328, Unigene11530, Unigene537, Unigene54734, Unigene10541, CL359.Contig1, Unigene8272, Unigene9775, Unigene53958, Unigene52323, Unigene10694, Unigene32831, Unigene52289, Unigene10008, CL2431.Contig3, Unigene19560, CL4159.Contig2, Unigene52338, Unigene52524, Unigene19739, Unigene19577, Unigene10095, Unigene44323, Unigene52263, Unigene52614, Unigene53503, Unigene40246, Unigene23918, Unigene42146, CL3841.Contig1, Unigene10060, Unigene52808, CL2943.Contig2, Unigene32921, Unigene10288, CL4655.Contig1, Unigene55020, Unigene52179, Unigene9917, Unigene20876, Unigene31648, CL3555.Contig2, Unigene9620, Unigene15125, Unigene39276, Unigene10973, Unigene52313, Unigene52557, Unigene52636, Unigene39931, Unigene53050, Unigene53878, Unigene16329, Unigene18792, Unigene39553, Unigene52334, CL910.Contig2, Unigene10261, Unigene10195, Unigene30673, Unigene52447, Unigene34361, Unigene30980, Unigene33870, Unigene52515, Unigene29212, Unigene52905, Unigene32094, Unigene48898, Unigene38188, CL3725.Contig2, Unigene52336, Unigene48738, Unigene10146, Unigene52409,	**180**

oxoacid metabolic process	Unigene24445, Unigene30808, Unigene33006, Unigene15125, Unigene39276, Unigene99, Unigene39521, Unigene22431, Unigene19978, Unigene53878, Unigene52381, Unigene23422, CL167.Contig2, Unigene39553, Unigene32831, Unigene34361, Unigene27336, Unigene25581, Unigene52289, Unigene30918, Unigene33606, Unigene32094, Unigene38849, Unigene22035, Unigene53503, Unigene40246, Unigene49671, Unigene52649, CL3398.Contig1, Unigene19323, Unigene23918, Unigene52769, Unigene52279, CL3845.Contig2, Unigene54755	**35**
organic acid metabolic process	Unigene24445, Unigene30808, Unigene33006, Unigene15125, Unigene39276, Unigene99, Unigene39521, Unigene22431, Unigene19978, Unigene53878, Unigene52381, Unigene23422, CL167.Contig2, Unigene39553, Unigene32831, Unigene34361, Unigene27336, Unigene25581, Unigene52289, Unigene30918, Unigene33606, Unigene32094, Unigene38849, Unigene22035, Unigene53503, Unigene40246, Unigene49671, Unigene52649, CL3398.Contig1, Unigene19323, Unigene23918, Unigene52769, Unigene52279, CL3845.Contig2, Unigene54755	**35**
biosynthetic process	Unigene10079, CL990.Contig2, Unigene30808, Unigene52324, Unigene30706, Unigene52328, Unigene52732, Unigene39521, Unigene22431, Unigene10541, CL4794.Contig1, CL359.Contig1, Unigene51321, Unigene52323, Unigene10694, Unigene32831, Unigene27336, Unigene25581, Unigene52289, CL2431.Contig3, Unigene52338, CL4159.Contig2, Unigene52644, Unigene30135, Unigene22035, Unigene52562, Unigene44323, Unigene52614, Unigene53503, Unigene40246, CL3398.Contig1, Unigene23918, Unigene42146, Unigene52769, Unigene52590, CL2943.Contig2, Unigene32921, Unigene54755, CL4655.Contig1, Unigene52179, Unigene29233, Unigene24445, Unigene33331, Unigene9620, Unigene15125, Unigene99, Unigene10973, Unigene28586, Unigene52313, Unigene52636, Unigene53100, Unigene52435, Unigene16329, Unigene52334, CL910.Contig2, Unigene10195, Unigene30673, Unigene52447, Unigene34361, CL240.Contig1, Unigene33259, Unigene30918, Unigene33870, Unigene29212, Unigene20116, Unigene33606, Unigene9627, Unigene52336, Unigene52332, Unigene32069, Unigene10146, Unigene20206, Unigene34417, Unigene52409, Unigene49671, Unigene11676, Unigene11765, Unigene9952, Unigene52279, CL5144.Contig2, Unigene10025	**81**
protein targeting	CL990.Contig2, Unigene23670, Unigene10095, Unigene52332, CL910.Contig2, Unigene53378, Unigene52764, Unigene10195, Unigene9620, Unigene30706, Unigene52447, Unigene52590, CL2431.Contig3, Unigene52338	**14**
small molecule metabolic process	Unigene24445, Unigene30808, Unigene33006, Unigene8259, Unigene15125, Unigene39276, Unigene99, Unigene11530, Unigene52810, Unigene53895, Unigene32952, Unigene39521, Unigene28586, Unigene22431, Unigene52557, Unigene52636, Unigene39931, Unigene10541, CL4794.Contig1, Unigene19978, Unigene53878, Unigene52381, CL359.Contig1, Unigene23422, Unigene16329, CL167.Contig2, Unigene39553, Unigene30673, Unigene32831, Unigene34361, Unigene27336, CL240.Contig1, Unigene25581, Unigene52289, Unigene33870, Unigene30918, Unigene16177, Unigene10378, Unigene33606, Unigene32094, Unigene38849, Unigene22035, Unigene53503, Unigene42147, Unigene29431, Unigene40246, Unigene49671, Unigene52649, CL3398.Contig1, Unigene23918, Unigene19323, Unigene42146, Unigene52769, Unigene31292, Unigene46198, Unigene52279, Unigene10060, Unigene10649, CL3845.Contig2, Unigene32921, Unigene54755, Unigene29432, Unigene29233	**63**
cellular component disassembly	CL990.Contig2, Unigene52332, CL910.Contig2, Unigene10195, Unigene9620, Unigene52447, Unigene30706, Unigene52590, CL2431.Contig3, Unigene52338	**10**
cellular biosynthetic process	Unigene10079, CL990.Contig2, Unigene52324, Unigene30706, Unigene52328, Unigene52732, Unigene39521, Unigene22431, Unigene10541, CL4794.Contig1, CL359.Contig1, Unigene52323, Unigene10694, Unigene32831, Unigene27336, Unigene25581, Unigene52289, CL2431.Contig3, Unigene52338, CL4159.Contig2, Unigene52644, Unigene30135, Unigene22035, Unigene52562, Unigene44323, Unigene52614, Unigene53503, Unigene40246, CL3398.Contig1, Unigene23918, Unigene42146, Unigene52769, Unigene52590, CL2943.Contig2, Unigene32921, Unigene54755, CL4655.Contig1, Unigene52179, Unigene29233, Unigene24445, Unigene33331, Unigene9620, Unigene15125, Unigene99, Unigene10973, Unigene28586, Unigene52313, Unigene16329, Unigene52334, CL910.Contig2, Unigene10195, Unigene30673, Unigene52447, Unigene34361, Unigene33259, CL240.Contig1, Unigene30918, Unigene33870, Unigene29212, Unigene33606, Unigene9627, Unigene52332, Unigene32069, Unigene10146, Unigene20206, Unigene34417, Unigene52409, Unigene49671, Unigene11676, Unigene11765, Unigene9952, Unigene52279, CL5144.Contig2, Unigene10025	**74**

monosaccharide biosynthetic process	Unigene52769, Unigene30808, Unigene30918, Unigene51321, Unigene52336, Unigene52636, Unigene53100	**7**
nitrogen compound metabolic process	Unigene52327, Unigene33668, CL990.Contig2, Unigene28660, Unigene9001, Unigene12267, Unigene8259, Unigene30706, Unigene11530, Unigene52810, Unigene52732, Unigene537, Unigene54734, Unigene33552, Unigene10541, CL4794.Contig1, Unigene19978, Unigene9670, CL359.Contig1, Unigene23422, Unigene8272, Unigene9775, Unigene53958, Unigene27336, Unigene25581, Unigene52289, CL2431.Contig3, Unigene19560, Unigene52338, Unigene52644, Unigene38849, Unigene22035, Unigene33439, Unigene53503, Unigene42147, Unigene40246, Unigene9179, CL3398.Contig1, Unigene11495, CL112.Contig1, Unigene10041, Unigene23918, Unigene42146, Unigene52769, Unigene31292, Unigene8404, Unigene52590, Unigene52808, Unigene10060, CL2943.Contig2, Unigene32921, Unigene16063, Unigene54755, Unigene52179, Unigene29233, Unigene24445, Unigene9917, CL3555.Contig2, Unigene53320, Unigene9620, Unigene15125, Unigene99, Unigene10973, Unigene28586, Unigene52557, Unigene16133, Unigene39931, Unigene52435, Unigene52381, Unigene16329, Unigene39553, Unigene10654, CL910.Contig2, Unigene28926, Unigene10261, Unigene10195, Unigene52667, Unigene30673, Unigene52447, Unigene34361, Unigene30980, CL240.Contig1, Unigene30918, Unigene33870, Unigene29212, Unigene52905, Unigene16177, Unigene46715, Unigene33606, Unigene32094, Unigene38188, Unigene52332, CL3725.Contig2, Unigene34417, Unigene29431, Unigene49671, Unigene10190, Unigene33311, Unigene11676, Unigene11765, Unigene52279, Unigene33507, CL3235.Contig1, Unigene10649, CL3845.Contig2, Unigene29432, CL5144.Contig2, Unigene1020	**108**
nuclear-transcribed mRNA catabolic process	CL990.Contig2, Unigene52332, CL910.Contig2, Unigene10195, Unigene9620, Unigene52447, Unigene30706, Unigene52590, CL2431.Contig3, Unigene52338	**10**
nematode larval development	Unigene29924, Unigene33311, CL1106.Contig1, Unigene10146, Unigene52592, Unigene42147, Unigene10649, Unigene10008, Unigene29432, CL5144.Contig2, Unigene54734, CL4655.Contig1	**12**
response to interferon-gamma	CL3398.Contig1, Unigene52336, Unigene52764	**3**
cellular amino acid metabolic process	Unigene33606, Unigene24445, Unigene32094, Unigene38849, Unigene22035, Unigene15125, Unigene99, Unigene53503, Unigene40246, Unigene49671, Unigene19978, Unigene52381, CL3398.Contig1, Unigene39553, Unigene23918, Unigene52769, Unigene34361, Unigene27336, Unigene52279, Unigene25581, Unigene52289, Unigene30918, CL3845.Contig2, Unigene54755	**24**
gluconeogenesis	Unigene52769, Unigene30808, Unigene51321, Unigene52336, Unigene52636, Unigene53100	**6**
hexose biosynthetic process	Unigene52769, Unigene30808, Unigene51321, Unigene52336, Unigene52636, Unigene53100	**6**
positive regulation of growth rate	Unigene20857, Unigene29924, Unigene33311, Unigene10146, Unigene42147, Unigene10008, Unigene22317, Unigene54734, CL4655.Contig1	**9**
ribosome	Unigene10079, CL990.Contig2, Unigene10358, Unigene52324, Unigene9620, Unigene30706, Unigene52328, Unigene52313, Unigene33750, Unigene52334, CL910.Contig2, Unigene52323, Unigene10195, Unigene10694, Unigene52447, CL2431.Contig3, Unigene29212, CL4159.Contig2, Unigene52424, Unigene52338, Unigene30501, Unigene9627, Unigene52332, Unigene52562, Unigene10146, Unigene20206, Unigene52409, Unigene52590, Unigene1020, CL4655.Contig1, CL5144.Contig2, Unigene10025	**32**

cytoplasm	Unigene23711, Unigene10079, CL990.Contig2, Unigene10358, Unigene52324, Unigene46231, CL5309.Contig1, Unigene30706, Unigene52732, Unigene8237, CL4794.Contig1, Unigene20857, Unigene23422, Unigene33750, Unigene51321, Unigene52373, Unigene52424, Unigene29924, Unigene10558, Unigene38849, Unigene22035, Unigene52562, Unigene42147, Unigene10034, CL110.Contig1, Unigene14695, CL3398.Contig1, Unigene10751, CL110.Contig2, Unigene52769, Unigene52590, Unigene52976, Unigene54755, Unigene473, Unigene29233, Unigene20593, Unigene22778, Unigene10823, Unigene99, Unigene53617, Unigene52435, Unigene53100, Unigene10312, Unigene28926, Unigene10007, CL3211.Contig2, Unigene30918, Unigene20116, Unigene48142, Unigene30501, Unigene9627, Unigene8502, Unigene33606, Unigene52332, Unigene32069, Unigene20206, Unigene55198, Unigene52649, Unigene19323, Unigene10649, Unigene1020, Unigene52838, Unigene30808, Unigene11530, Unigene52328, Unigene32952, Unigene52308, Unigene22431, Unigene537, Unigene54734, Unigene401, CL359.Contig1, Unigene23670, Unigene52323, Unigene10694, Unigene32831, Unigene10008, CL2431.Contig3, CL4159.Contig2, Unigene52338, Unigene52524, Unigene10520, Unigene10095, Unigene19577, Unigene44323, Unigene52319, Unigene52614, Unigene9773, Unigene53562, Unigene42146, Unigene10060, Unigene23095, CL2785.Contig1, CL2943.Contig2, Unigene29625, CL4655.Contig1, Unigene55020, Unigene52179, Unigene31648, Unigene52797, Unigene18083, Unigene9620, Unigene15125, Unigene52313, Unigene32162, Unigene52557, Unigene52636, Unigene39931, Unigene53050, Unigene53878, Unigene18792, Unigene52334, CL910.Contig2, Unigene52546, Unigene10195, Unigene30673, Unigene52447, Unigene15108, Unigene9599, Unigene33870, Unigene10808, Unigene52515, Unigene29212, Unigene32094, Unigene48898, Unigene38248, Unigene53860, Unigene52336, Unigene52764, Unigene10146, Unigene52409, Unigene52768, Unigene28552, Unigene29431, Unigene42992, Unigene10190, Unigene11267, Unigene46682, Unigene53034, CL3845.Contig2, CL2540.Contig1, Unigene29432, CL5144.Contig2, Unigene10025, Unigene39244	**145**
ribosomal subunit	Unigene9627, Unigene10079, CL990.Contig2, Unigene10358, Unigene52324, Unigene52332, Unigene9620, Unigene30706, Unigene52409, Unigene33750, Unigene52334, CL910.Contig2, Unigene52323, Unigene10195, Unigene52447, Unigene52590, CL2431.Contig3, Unigene29212, Unigene1020, Unigene52424, Unigene52338, CL5144.Contig2, Unigene10025	**23**
cytosolic ribosome	Unigene10079, CL990.Contig2, Unigene52332, Unigene9620, Unigene30706, Unigene20206, CL910.Contig2, Unigene52334, Unigene52323, Unigene10195, Unigene52447, Unigene52590, CL2431.Contig3, Unigene29212, Unigene52424, Unigene52338, CL5144.Contig2	**17**
cytoplasmic part	Unigene23711, Unigene10079, CL990.Contig2, Unigene30808, Unigene10358, Unigene52324, Unigene46231, CL5309.Contig1, Unigene30706, Unigene52328, Unigene11530, Unigene52732, Unigene32952, Unigene537, Unigene8237, Unigene54734, CL4794.Contig1, Unigene20857, Unigene401, CL359.Contig1, Unigene23422, Unigene33750, Unigene23670, Unigene51321, Unigene52323, Unigene10694, Unigene32831, CL2431.Contig3, Unigene10008, Unigene52338, Unigene52424, CL4159.Contig2, Unigene52524, Unigene10558, Unigene10520, Unigene38849, Unigene19577, Unigene10095, Unigene22035, Unigene52562, Unigene52319, Unigene44323, Unigene52614, Unigene9773, Unigene42147, Unigene10034, CL110.Contig1, Unigene53562, Unigene14695, CL3398.Contig1, CL110.Contig2, Unigene42146, Unigene52769, Unigene52590, Unigene23095, CL2785.Contig1, Unigene52976, Unigene29625, CL4655.Contig1, Unigene473, Unigene52179, Unigene29233, Unigene55020, Unigene20593, Unigene22778, Unigene10823, Unigene18083, Unigene52797, Unigene9620, Unigene15125, Unigene99, Unigene53617, Unigene52313, Unigene32162, Unigene52557, Unigene52636, Unigene52435, Unigene53100, Unigene53878, Unigene52334, Unigene52546, CL910.Contig2, Unigene10312, Unigene28926, Unigene10195, Unigene52447, Unigene10808, Unigene30918, Unigene33870, Unigene52515, Unigene29212, Unigene20116, Unigene48142, Unigene30501, Unigene9627, Unigene8502, Unigene33606, Unigene32094, Unigene38248, Unigene53860, Unigene52332, Unigene52336, Unigene52764, Unigene10146, Unigene20206, Unigene52409, Unigene52768, Unigene28552, Unigene42992, Unigene10190, Unigene55198, Unigene52649, Unigene11267, Unigene19323, Unigene53034, Unigene10649, CL3845.Contig2, CL5144.Contig2, Unigene1020, Unigene10025, Unigene52838	**121**
cytosolic part	Unigene10079, CL990.Contig2, Unigene52332, Unigene9620, Unigene30706, Unigene20206, Unigene52768, Unigene52334, CL910.Contig2, Unigene52323, Unigene10195, Unigene52447, Unigene52590, CL2431.Contig3, Unigene29212, Unigene52424, Unigene52338, CL5144.Contig2, Unigene52838	**19**

cytosol	Unigene10079, CL990.Contig2, Unigene10823, Unigene9620, Unigene30706, Unigene52732, Unigene537, Unigene52636, Unigene52435, CL4794.Contig1, CL359.Contig1, Unigene52334, CL910.Contig2, Unigene52323, Unigene28926, Unigene10195, Unigene52447, Unigene30918, CL2431.Contig3, Unigene29212, Unigene52338, Unigene52424, Unigene33606, Unigene32094, Unigene52336, Unigene52332, Unigene22035, Unigene52319, Unigene20206, Unigene10034, Unigene52768, Unigene10190, Unigene42146, Unigene52769, Unigene52590, Unigene53034, Unigene473, CL5144.Contig2, Unigene52838	**39**
small ribosomal subunit	Unigene9627, Unigene10079, CL990.Contig2, Unigene52334, Unigene52324, Unigene52332, Unigene52323, Unigene52447, Unigene52590, Unigene29212, CL5144.Contig2, Unigene52424, Unigene1020, Unigene52338, Unigene10025	**15**
mitochondrion	Unigene30808, Unigene20593, Unigene46231, Unigene18083, CL5309.Contig1, Unigene15125, Unigene99, Unigene53617, Unigene32952, Unigene52557, Unigene32162, Unigene54734, Unigene52435, Unigene401, Unigene53878, Unigene23422, Unigene23670, Unigene51321, Unigene10312, Unigene32831, Unigene10008, Unigene33870, Unigene52515, Unigene20116, Unigene33606, Unigene8502, Unigene52524, Unigene38849, Unigene10520, Unigene10095, Unigene53860, Unigene52336, Unigene22035, Unigene52614, Unigene9773, Unigene10034, Unigene28552, Unigene42992, Unigene10190, Unigene14695, Unigene53562, Unigene52649, CL3398.Contig1, Unigene11267, Unigene19323, Unigene23095, CL3845.Contig2, Unigene29625, Unigene52838	**49**
ribonucleoprotein complex	Unigene23711, Unigene10079, CL990.Contig2, Unigene10358, Unigene52324, Unigene9620, Unigene30706, Unigene52328, Unigene52308, Unigene52313, Unigene537, Unigene54734, Unigene12396, Unigene33750, Unigene52334, CL910.Contig2, Unigene52323, Unigene10195, Unigene10694, Unigene30673, Unigene52447, Unigene9604, Unigene9599, CL2431.Contig3, Unigene28563, Unigene29212, Unigene52338, CL4159.Contig2, Unigene52424, Unigene30501, Unigene9627, Unigene52336, Unigene52332, Unigene52562, Unigene10146, Unigene20206, Unigene34417, Unigene52409, Unigene10190, Unigene52590, Unigene1020, CL4655.Contig1, CL5144.Contig2, Unigene10025	**44**
cytosolic small ribosomal subunit	Unigene10079, CL990.Contig2, Unigene52334, Unigene52332, Unigene52323, Unigene52447, Unigene52590, Unigene29212, Unigene52424, Unigene52338, CL5144.Contig2	**11**
intracellular part	Unigene23711, Unigene10079, CL990.Contig2, Unigene10358, Unigene52324, Unigene46231, Unigene22590, CL5309.Contig1, Unigene30706, Unigene52732, CL5033.Contig2, Unigene8237, CL4794.Contig1, Unigene20857, Unigene23422, Unigene33750, Unigene51321, Unigene51842, Unigene52373, Unigene9604, Unigene10879, Unigene52424, Unigene29924, Unigene10558, Unigene38849, Unigene9290, Unigene22035, Unigene52562, Unigene42147, Unigene10034, CL110.Contig1, Unigene14695, CL3398.Contig1, Unigene10751, CL110.Contig2, Unigene52769, Unigene31292, Unigene52590, Unigene52976, Unigene54755, Unigene473, Unigene29233, Unigene20593, Unigene22778, Unigene10823, Unigene99, Unigene52778, Unigene53617, Unigene9697, Unigene15367, Unigene52435, Unigene53100, Unigene10312, Unigene28926, Unigene10007, CL3211.Contig2, CL240.Contig1, CL650.Contig2, Unigene30918, Unigene20116, Unigene28563, Unigene48142, Unigene30501, Unigene9627, Unigene8502, Unigene33606, Unigene52332, Unigene32069, Unigene20206, Unigene34417, Unigene21269, Unigene55198, Unigene33311, Unigene11676, Unigene52649, Unigene19323, Unigene10649, Unigene10719, Unigene1020, Unigene52838, Unigene30808, Unigene43286, Unigene52328, Unigene11530, Unigene32952, Unigene52308, Unigene22431, Unigene537, Unigene54734, Unigene401, CL359.Contig1, Unigene23670, Unigene52323, Unigene10694, Unigene32831, Unigene10008, CL2431.Contig3, CL4159.Contig2, Unigene52338, Unigene52524, Unigene10520, Unigene19739, Unigene19577, Unigene10095, Unigene44323, Unigene52319, Unigene52614, Unigene9773, Unigene53562, Unigene42146, Unigene10060, Unigene23095, CL2785.Contig1, CL2943.Contig2, Unigene32921, Unigene29625, Unigene10288, CL4655.Contig1, Unigene55020, Unigene52179, Unigene38804, Unigene31648, Unigene33248, Unigene52797, Unigene18083, CL3555.Contig2, Unigene9620, Unigene15125, Unigene10973, Unigene52313, Unigene32162, Unigene52557, Unigene52636, Unigene39931, Unigene53050, Unigene12396, Unigene53878, Unigene18792, Unigene52334, CL910.Contig2, Unigene52878, Unigene52546, Unigene10195, Unigene30673, Unigene52447, Unigene33743, Unigene15108, Unigene9599, Unigene33870, Unigene10808, Unigene52515, Unigene29212, Unigene32094, Unigene48898, Unigene38248, Unigene53860, Unigene52336, Unigene52764, Unigene10146, Unigene52409, Unigene52768, Unigene28552, Unigene29431, Unigene42992, Unigene10190, Unigene9488, Unigene11267, Unigene34497, Unigene46682, Unigene53034, CL2540.Contig1, CL3845.Contig2, Unigene53261, Unigene29432, CL5144.Contig2, Unigene10025, Unigene39244	**177**

intracellular	Unigene23711, Unigene10079, CL990.Contig2, Unigene10358, Unigene52324, Unigene46231, Unigene52410, Unigene22590, CL5309.Contig1, Unigene30706, Unigene52732, CL5033.Contig2, Unigene8237, CL4794.Contig1, Unigene20857, Unigene23422, Unigene33750, Unigene51321, Unigene51842, Unigene52373, Unigene9604, Unigene10879, Unigene52424, Unigene29924, Unigene10558, Unigene38849, Unigene52804, Unigene9290, Unigene22035, Unigene52562, Unigene42147, Unigene10034, CL110.Contig1, Unigene14695, CL3398.Contig1, Unigene10751, CL110.Contig2, Unigene52769, Unigene31292, Unigene52590, Unigene52976, Unigene54755, Unigene473, Unigene29233, Unigene20593, Unigene22778, Unigene10823, Unigene99, Unigene52778, Unigene53617, Unigene9697, Unigene15367, Unigene52435, Unigene53100, Unigene10312, Unigene28926, Unigene10007, CL3211.Contig2, CL240.Contig1, CL650.Contig2, Unigene30918, Unigene20116, Unigene28563, Unigene48142, Unigene30501, Unigene9627, Unigene8502, Unigene33606, Unigene52332, Unigene32069, Unigene20206, Unigene34417, Unigene21269, Unigene43948, Unigene55198, Unigene33311, Unigene11676, Unigene52649, Unigene19323, Unigene10649, Unigene10719, Unigene1020, Unigene52838, Unigene30808, Unigene43286, Unigene52328, Unigene11530, Unigene32952, Unigene52308, Unigene22431, Unigene537, Unigene54734, Unigene401, Unigene52426, CL359.Contig1, Unigene23670, Unigene52323, Unigene22721, Unigene10694, Unigene32831, Unigene10008, CL2431.Contig3, CL4159.Contig2, Unigene52338, Unigene52524, Unigene10520, Unigene19739, Unigene19577, Unigene10095, Unigene52319, Unigene44323, Unigene52614, Unigene9773, Unigene53562, Unigene42146, Unigene10060, Unigene23095, CL2785.Contig1, CL2943.Contig2, Unigene32921, Unigene29625, Unigene52549, Unigene10288, CL4655.Contig1, Unigene55020, Unigene52179, Unigene38804, Unigene31648, Unigene33248, Unigene52797, Unigene18083, CL3555.Contig2, Unigene9620, Unigene15125, Unigene10973, Unigene52313, Unigene32162, Unigene52557, Unigene52636, Unigene39931, Unigene53050, Unigene12396, Unigene53878, Unigene18792, Unigene52334, CL910.Contig2, Unigene52878, Unigene52546, Unigene10195, Unigene30673, Unigene52447, Unigene33743, Unigene15108, Unigene9599, Unigene33870, Unigene10808, Unigene52515, Unigene29212, Unigene32094, Unigene48898, Unigene38248, Unigene53860, Unigene52336, Unigene52764, Unigene10146, Unigene52409, Unigene52768, Unigene28552, Unigene29431, Unigene42992, Unigene10190, Unigene9488, Unigene11267, Unigene34497, Unigene46682, Unigene53034, CL2540.Contig1, CL3845.Contig2, Unigene53261, Unigene29432, CL5144.Contig2, Unigene10025, Unigene39244	**183**
mitochondrial part	Unigene52524, Unigene30808, Unigene10520, Unigene38849, Unigene20593, Unigene10095, Unigene53860, Unigene46231, Unigene18083, Unigene52614, CL5309.Contig1, Unigene32952, Unigene52557, Unigene401, Unigene53878, Unigene23422, Unigene53562, Unigene52649, CL3398.Contig1, Unigene11267, Unigene10312, Unigene33870, CL3845.Contig2	**23**
large ribosomal subunit	Unigene10195, Unigene9620, Unigene30706, Unigene33750, Unigene10358, Unigene52409, CL2431.Contig3, CL910.Contig2	**8**
proteasome core complex	Unigene22590, Unigene10190, Unigene31648, Unigene10879, Unigene10288, Unigene28926	**6**
proteasome complex	Unigene22590, Unigene10190, Unigene31648, Unigene10879, Unigene33248, Unigene10288, Unigene53050, Unigene28926, Unigene42146	**9**
cytosolic large ribosomal subunit	Unigene10195, Unigene9620, Unigene30706, CL2431.Contig3, CL910.Contig2	**5**

intracellular organelle	Unigene23711, Unigene10079, CL990.Contig2, Unigene10358, Unigene52324, Unigene46231, CL5309.Contig1, Unigene30706, Unigene52732, CL5033.Contig2, Unigene8237, CL4794.Contig1, Unigene20857, Unigene23422, Unigene33750, Unigene51321, Unigene51842, Unigene52373, Unigene52424, Unigene29924, Unigene10558, Unigene38849, Unigene9290, Unigene22035, Unigene52562, Unigene42147, Unigene10034, CL110.Contig1, Unigene14695, CL3398.Contig1, CL110.Contig2, Unigene31292, Unigene52590, Unigene52976, Unigene473, Unigene29233, Unigene20593, Unigene22778, Unigene10823, Unigene99, Unigene52778, Unigene53617, Unigene9697, Unigene52435, Unigene10312, Unigene28926, CL3211.Contig2, CL650.Contig2, Unigene30918, Unigene20116, Unigene48142, Unigene30501, Unigene9627, Unigene8502, Unigene33606, Unigene52332, Unigene20206, Unigene55198, Unigene33311, Unigene11676, Unigene52649, Unigene19323, Unigene10649, Unigene1020, Unigene52838, Unigene30808, Unigene43286, Unigene11530, Unigene52328, Unigene32952, Unigene537, Unigene54734, Unigene401, Unigene23670, Unigene52323, Unigene10694, Unigene32831, Unigene10008, CL2431.Contig3, CL4159.Contig2, Unigene52338, Unigene52524, Unigene10520, Unigene10095, Unigene19577, Unigene19739, Unigene44323, Unigene52319, Unigene52614, Unigene9773, Unigene53562, Unigene42146, Unigene23095, CL2785.Contig1, CL2943.Contig2, Unigene29625, CL4655.Contig1, Unigene55020, Unigene52179, Unigene38804, Unigene31648, Unigene52797, Unigene18083, CL3555.Contig2, Unigene9620, Unigene15125, Unigene10973, Unigene52313, Unigene32162, Unigene52557, Unigene52636, Unigene39931, Unigene53050, Unigene53878, Unigene52334, CL910.Contig2, Unigene52546, Unigene10195, Unigene52447, Unigene33743, Unigene15108, Unigene33870, Unigene10808, Unigene52515, Unigene29212, Unigene48898, Unigene38248, Unigene53860, Unigene52336, Unigene52764, Unigene10146, Unigene52409, Unigene52768, Unigene28552, Unigene29431, Unigene42992, Unigene10190, Unigene11267, Unigene34497, CL3845.Contig2, CL2540.Contig1, Unigene53261, Unigene29432, CL5144.Contig2, Unigene10025, Unigene39244	**146**
cell part	Unigene23711, Unigene10079, CL990.Contig2, Unigene10358, Unigene52324, Unigene46231, Unigene52410, Unigene22590, CL5309.Contig1, Unigene30706, Unigene52732, CL5033.Contig2, Unigene8237, CL4794.Contig1, Unigene20857, Unigene23422, Unigene33750, Unigene51321, Unigene51842, Unigene52373, Unigene9604, Unigene10879, Unigene52424, Unigene29924, Unigene10558, Unigene38849, Unigene52804, Unigene9290, Unigene22035, Unigene52562, Unigene42147, Unigene10034, CL110.Contig1, Unigene14695, CL3398.Contig1, Unigene10751, CL110.Contig2, Unigene52769, Unigene31292, Unigene52590, Unigene52976, Unigene54755, Unigene473, Unigene29233, Unigene20593, Unigene22778, Unigene10823, Unigene52733, Unigene99, Unigene735, Unigene52778, Unigene53617, Unigene9697, Unigene15367, Unigene52435, Unigene53100, Unigene10312, Unigene28926, Unigene10007, CL3211.Contig2, CL240.Contig1, CL650.Contig2, Unigene30918, Unigene20116, Unigene28563, Unigene48142, Unigene30501, Unigene9627, Unigene8502, Unigene33606, Unigene52332, Unigene32069, Unigene34428, Unigene20206, Unigene34417, Unigene21269, Unigene43948, Unigene55198, Unigene33311, Unigene11676, Unigene52649, Unigene19323, Unigene10649, Unigene10719, Unigene1020, Unigene52838, Unigene30808, Unigene43286, Unigene52328, Unigene11530, Unigene32952, Unigene52308, Unigene22431, Unigene537, Unigene54734, Unigene401, Unigene52426, CL359.Contig1, Unigene23670, Unigene52323, Unigene22721, Unigene10694, Unigene32831, Unigene10008, CL2431.Contig3, CL4159.Contig2, Unigene52338, Unigene52524, Unigene10520, Unigene19739, Unigene19577, Unigene10095, Unigene52319, Unigene44323, Unigene52614, Unigene9773, Unigene34757, Unigene53562, CL1669.Contig3, Unigene42146, Unigene10060, Unigene23095, CL2785.Contig1, CL2943.Contig2, Unigene32921, CL4844.Contig2, Unigene29625, Unigene52549, Unigene10288, CL4655.Contig1, Unigene55020, Unigene52179, Unigene38804, Unigene31648, Unigene33248, Unigene52797, Unigene18083, CL3555.Contig2, Unigene9620, Unigene15125, Unigene10973, Unigene52313, Unigene32162, Unigene52557, Unigene52636, Unigene39931, Unigene53050, Unigene12396, Unigene53878, Unigene9095, Unigene18792, Unigene52334, CL910.Contig2, Unigene52878, Unigene52546, Unigene10195, Unigene30673, Unigene52447, Unigene33743, Unigene15108, Unigene9599, Unigene33870, Unigene10808, Unigene52515, Unigene29212, Unigene32094, Unigene48898, Unigene38248, Unigene53860, Unigene52336, Unigene52764, Unigene10146, Unigene52409, Unigene52768, Unigene28552, Unigene29431, Unigene42992, Unigene10190, Unigene9488, Unigene11267, Unigene34497, Unigene46682, Unigene53034, CL2540.Contig1, CL3845.Contig2, Unigene53261, Unigene29432, CL5144.Contig2, Unigene10025, Unigene39244	**190**

cell	Unigene23711, Unigene10079, CL990.Contig2, Unigene10358, Unigene52324, Unigene46231, Unigene52410, Unigene22590, CL5309.Contig1, Unigene30706, Unigene52732, CL5033.Contig2, Unigene8237, CL4794.Contig1, Unigene20857, Unigene23422, Unigene33750, Unigene51321, Unigene51842, Unigene52373, Unigene9604, Unigene10879, Unigene52424, Unigene29924, Unigene10558, Unigene38849, Unigene52804, Unigene9290, Unigene22035, Unigene52562, Unigene42147, Unigene10034, CL110.Contig1, Unigene14695, CL3398.Contig1, Unigene10751, CL110.Contig2, Unigene52769, Unigene31292, Unigene52590, Unigene52976, Unigene54755, Unigene473, Unigene29233, Unigene20593, Unigene22778, Unigene10823, Unigene52733, Unigene99, Unigene735, Unigene52778, Unigene53617, Unigene9697, Unigene15367, Unigene52435, Unigene53100, Unigene10312, Unigene28926, Unigene10007, CL3211.Contig2, CL240.Contig1, CL650.Contig2, Unigene30918, Unigene20116, Unigene28563, Unigene48142, Unigene30501, Unigene9627, Unigene8502, Unigene33606, Unigene52332, Unigene32069, Unigene34428, Unigene20206, Unigene34417, Unigene21269, Unigene43948, Unigene55198, Unigene33311, Unigene11676, Unigene52649, Unigene19323, Unigene10649, Unigene10719, Unigene1020, Unigene52838, Unigene30808, Unigene43286, Unigene52328, Unigene11530, Unigene32952, Unigene52308, Unigene22431, Unigene537, Unigene54734, Unigene401, Unigene52426, CL359.Contig1, Unigene23670, Unigene52323, Unigene22721, Unigene10694, Unigene32831, Unigene10008, CL2431.Contig3, CL4159.Contig2, Unigene52338, Unigene52524, Unigene10520, Unigene19739, Unigene19577, Unigene10095, Unigene52319, Unigene44323, Unigene52614, Unigene9773, Unigene34757, Unigene53562, CL1669.Contig3, Unigene42146, Unigene10060, Unigene23095, CL2785.Contig1, CL2943.Contig2, Unigene32921, CL4844.Contig2, Unigene29625, Unigene52549, Unigene10288, CL4655.Contig1, Unigene55020, Unigene52179, Unigene38804, Unigene31648, Unigene33248, Unigene52797, Unigene18083, CL3555.Contig2, Unigene9620, Unigene15125, Unigene10973, Unigene52313, Unigene32162, Unigene52557, Unigene52636, Unigene39931, Unigene53050, Unigene12396, Unigene53878, Unigene9095, Unigene18792, Unigene52334, CL910.Contig2, Unigene52878, Unigene52546, Unigene10195, Unigene30673, Unigene52447, Unigene33743, Unigene15108, Unigene9599, Unigene33870, Unigene10808, Unigene52515, Unigene29212, Unigene32094, Unigene48898, Unigene38248, Unigene53860, Unigene52336, Unigene52764, Unigene10146, Unigene52409, Unigene52768, Unigene28552, Unigene29431, Unigene42992, Unigene10190, Unigene9488, Unigene11267, Unigene34497, Unigene46682, Unigene53034, CL2540.Contig1, CL3845.Contig2, Unigene53261, Unigene29432, CL5144.Contig2, Unigene10025, Unigene39244	**190**
polysome	Unigene30673, Unigene10190, CL990.Contig2, Unigene20206	**4**
structural constituent of ribosome	Unigene9627, Unigene10079, CL990.Contig2, Unigene10358, Unigene52324, Unigene52332, Unigene52562, Unigene10146, Unigene9620, Unigene52328, Unigene52409, Unigene52313, Unigene52334, CL910.Contig2, Unigene52323, Unigene10195, Unigene10694, Unigene52447, Unigene52590, CL2431.Contig3, Unigene29212, CL5144.Contig2, CL4159.Contig2, Unigene52338, CL4655.Contig1, Unigene10025	**26**
structural molecule activity	Unigene52889, Unigene10079, CL990.Contig2, Unigene10358, Unigene52324, CL2516.Contig2, Unigene9620, Unigene47692, Unigene52328, Unigene41899, Unigene52313, Unigene39931, Unigene52435, Unigene52334, CL910.Contig2, Unigene52323, Unigene10195, Unigene10694, CL2948.Contig2, Unigene52447, CL2431.Contig3, Unigene29212, CL4159.Contig2, Unigene52338, Unigene9627, Unigene52332, Unigene52562, Unigene10146, Unigene52409, Unigene29431, CL110.Contig1, Unigene49129, Unigene52590, CL4655.Contig1, CL5144.Contig2, Unigene29432, Unigene10025	**37**

Supplementary Table S3: RT-qPCR comfirmation results

Gene ID	Annotation/function	Method	Log_2Ratio(A/B)				
			V25/N25	N5/N25	N36/N25	V5/N5	V36/N36
CL1432.Contig1	intestinal mucin-2; cuticular protein 6 precursor	RNA-Seq	3.42	4.66	4.39	*-0.31*	-0.10
		RT-qPCR	3.88	4.79	4.12	*0.25*	-0.57
Unigene49129	similar to cuticular protein 62Bc	RNA-Seq	2.06	3.09	1.73	-1.06	1.14
		RT-qPCR	1.87	2.64	1.39	-1.42	0.92
Unigene908	E3 ubiquitin-protein ligase LRSAM1	RNA-Seq	1.35	*0.52*	1.13	1.41	1.74
		RT-qPCR	2.11	*-0.44*	1.65	1.82	2.33
Unigene43286	Mucosal immune response; stress-activated MAPK cascade	RNA-Seq	0.74	-0.54	*0.34*	1.76	1.15
		RT-qPCR	0.50	-0.22	*-0.17*	1.36	0.93
Unigene25547	RNA-induced silencing complex	RNA-Seq	*-0.05*	-0.31	-0.64	*-0.38*	-1.93

Unigene25547	RNA-induced silencing complex	RT-qPCR	*0.82*	-0.81	-0.31	*0.85*	-0.98
Unigene52300	cytochrome P450	RNA-Seq	2.88	1.76	2.30	*0.87*	-0.42
		RT-qPCR	3.27	1.82	2.77	*-0.35*	-0.94
Unigene8341	heat shock protein 78	RNA-Seq	0.83	*-0.47*	3.89	*-0.52*	-0.72
		RT-qPCR	1.13	*0.37*	4.69	*0.39*	-0.67
Unigene9618	heat shock protein 70	RNA-Seq	-0.05	-0.47	1.14	1.02	0.65
		RT-qPCR	-0.08	-0.36	0.93	1.78	0.57
Unigene16177	heat shock protein 70	RNA-Seq	-0.05	-0.33	-0.76	-0.36	-1.54
		RT-qPCR	-0.38	-0.52	-0.63	-1.04	-1.85
Unigene9991	heat shock protein 20	RNA-Seq	0.49	1.08	1.58	*-0.84*	-1.23
		RT-qPCR	1.19	0.54	0.16	*0.46*	-1.11
Unigene47833	putative small heat shock protein	RNA-Seq	1.34	0.82	4.24	*-0.28*	0.63
		RT-qPCR	2.01	1.34	4.99	*0.55*	0.56
Unigene39864	facilitated trehalose transporter Tret1-like	RNA-Seq	*-0.58*	-1.99	-0.04	1.44	0.95
		RT-qPCR	*0.04*	-1.72	-0.42	2.08	0.31

Supplementary Table S4: RT-qPCT primers used in this study

Gene ID	Forward primer (5' to 3')	Reverse primer (5' to 3')
CL1432.Contig1	TGACCACCGGCTACCCCTCT	CGTCGACGTGACCGTGATGTC
Unigene49129	TCCTATCCGCAACAGGCAGATCA	CTGGTGGCTCCTGGTAGTCGA
Unigene908	ACTGAACTCAAGGGCTGCAATCTG	GCTCATCTGGTTGTTTGACAGGTTCA
Unigene43286	GACAGGCATTCGGCGCTTCGA	TGTCTTCCCGGGTGGCTTCGA
Unigene25547	TACACTCATCGCATTGGCCGAAC	GGGATCTCTTGCTTGGTTTCCAG
Unigene52300	CTGCACGATTTACTGATGAGGTG	TGGCGACCAAGTAGATACAAGCAG
Unigene8341	GTTGCGGCTTGAGGTATCGAATGA	TCCACCAGCTCCACCATAGCTG
Unigene9618	ATGGCTGCGATGTCTGTGATAGG	TCGTTCGCAATGGTTTCTATGCCG
Unigene16177	GGCTGGACGCTGAGACGATCA	CGCAGACTACGCCAGGCATAGT
Unigene9991	CCTCTCTTCCGCCTGCTTGATG	CCATGTAGTTCATAGCTGTCTGACG
Unigene47833	GTGGTTATGTTCGTCCATGGAGG	GCTTGAACTGCTGGACATCCAG
Unigene39864	TCGAACAGTACACCGTACACTCTAG	TTGACCATGTAAGGCCTGAATCCAG
18S rRNA	AGTATCAATTGGAGGGCAAGTCTG	CACACAGTATACAGGCGTGACAAG
